# Design of a syringe extension device (Chloe SED®) for low-resource settings in sub-Saharan Africa: a circular economy approach

**DOI:** 10.3389/fmedt.2023.1183179

**Published:** 2023-09-01

**Authors:** Karlheinz Tondo Samenjo, Aparna Ramanathan, Stephen Otieno Gwer, Robert C. Bailey, Fredrick Odhiambo Otieno, Erin Koksal, Benjamin Sprecher, Rebecca Anne Price, Conny Bakker, Jan Carel Diehl

**Affiliations:** ^1^ Department of Sustainable Design Engineering, Faculty of Industrial Design Engineering, Delft University of Technology, Delft, Netherlands; ^2^ Nyanza Reproductive Health Society, Kisumu, Kenya; ^3^ Department of Obstetrics and Gynecology, National Center for Advanced Pelvic Surgery, Medstar Washington Hospital Center, Georgetown University, Washington, DC, United States; ^4^ Department of Obstetrics and Gynaecology, Maseno University, Kisumu, Kenya; ^5^ Division of Epidemiology and Biostatistics, School of Public Health, University of Illinois at Chicago, Chicago, IL, United States; ^6^ Rethink Robotics, Boston, MA, United States

**Keywords:** medical device design, context-driven design, circular economy, health and environment, low-resource settings, sub-Saharan Africa

## Abstract

Underfunded healthcare infrastructures in low-resource settings in sub-Saharan Africa have resulted in a lack of medical devices crucial to provide healthcare for all. A representative example of this scenario is medical devices to administer paracervical blocks during gynaecological procedures. Devices needed for this procedure are usually unavailable or expensive. Without these devices, providing paracervical blocks for women in need is impossible resulting in compromising the quality of care for women requiring gynaecological procedures such as loop electrosurgical excision, treatment of miscarriage, or incomplete abortion. In that perspective, interventions that can be integrated into the healthcare system in low-resource settings to provide women needing paracervical blocks remain urgent. Based on a context-specific approach while leveraging circular economy design principles, this research catalogues the development of a new medical device called Chloe SED® that can be used to support the provision of paracervical blocks. Chloe SED®, priced at US$ 1.5 per device when produced in polypropylene, US$ 10 in polyetheretherketone, and US$ 15 in aluminium, is attached to any 10-cc syringe in low-resource settings to provide paracervical blocks. The device is designed for durability, repairability, maintainability, upgradeability, and recyclability to address environmental sustainability issues in the healthcare domain. Achieving the design of Chloe SED® from a context-specific and circular economy approach revealed correlations between the material choice to manufacture the device, the device's initial cost, product durability and reuse cycle, reprocessing method and cost, and environmental impact. These correlations can be seen as interconnected conflicting or divergent trade-offs that need to be continually assessed to deliver a medical device that provides healthcare for all with limited environmental impact. The study findings are intended to be seen as efforts to make available medical devices to support women's access to reproductive health services.

## 1. Introduction

Over the past century, healthcare provision in low-resource settings (LRS) in sub-Saharan Africa (SSA) has been hampered by underfunded healthcare infrastructures (1–3). This results in a lack of medical devices crucial to provide healthcare for all (4, 5). Medical devices, which are used for a variety of purposes in the prevention, diagnosis, or treatment of illnesses or diseases, or to detect, measure, restore, and modify the body's structure for health purposes, are a vital component of any functioning healthcare system (6). However, these devices are expensive and often unaffordable to the healthcare systems in LRS (7). The high cost of medical devices has often resulted in LRS relying on international donations to equip healthcare facilities with medical devices. Estimates suggest that approximately 80% of medical device availability in healthcare facilities in LRS is covered by donations (8, 9). Presumably, this high volume of donations should drastically improve the availability of functioning medical devices in LRS (3). Nevertheless, these initiatives have been estimated to be unsuitable and unsustainable (3). Estimates suggest that about 40% of medical devices donated are non-functional, thus leaving healthcare facilities in LRS with the issue of medical device unavailability and excessive waste streams of defunct devices (10–12).

Philanthropic donations may help provide medical devices in LRS, but these initiatives are fraught with considerable limitations (13). For example, the non-prioritisation of essential medical devices has previously been highlighted as a significant limitation (10). Also, these donated medical devices are usually not optimised to function or operate in low-resource healthcare systems, coupled with the lack of trained personnel to use and maintain them (14). Donations also require long-term commitments to ensure the availability or continuous functioning of the device, but these are usually not provided or not sustained over time (15, 16). Other limitations include a lack of an adequate supply chain system, which prevents donated medical devices or consumables from reaching their intended users in local communities (17, 18). Ultimately, local communities in LRS remain with limited or no medical devices to provide healthcare for all. Representative for this scenario is the case of medical devices needed to provide women with a paracervical block (PCB) during gynaecological procedures.

PCB is a type of regional nerve block used to provide pain relief during gynaecological procedures (19, 20). It is performed by injecting an anaesthetic solution around the cervix to numb nearby nerves and reduce any discomfort (21). Examples of gynaecological procedures requiring PCB include loop electrosurgical excision procedure (LEEP), cervical biopsies, placement of contraceptives in the uterus, curettage, or manual vacuum aspiration (MVA) for the treatment of miscarriage or incomplete abortion (22–24). Neglecting PCB unnecessarily increases anxiety and pain, and compromises the quality of care for women requiring gynaecological procedures (25). However, providing this procedure is often difficult or impossible without access to the proper medical device.

Medical devices used to provide PCB include syringes attached to long enough needles to inject 20 ml of 1% lidocaine or 10 ml of 2% lidocaine to a depth of 3 cm in the cervix (26, 27). Examples of such needles include 20 gauge by 130-mm-long pudendal block needles (28), standard or extended-length spinal needles, or needle extenders. Although these syringe needles are commonplace medical devices in medical facilities, they are often unavailable in LRS (29, 30). When available, the prices can range between US$ 1.5 and 28 per needle (31–34). These prices can be high for LRS, particularly for those at the average poverty line of US$ 1.25 per day (35).

Philanthropic initiatives, such as the United States Agency for International Development (USAID) Post Abortion Care program (36) and Pathfinder International's Youth-Friendly Postabortion Care Project (37), are at the forefront of providing women in LRS in SSA with PCB-related procedures. As mentioned, relying on philanthropic initiatives is fraught with limitations, especially with the growing healthcare demand caused by an increasing African population (38, 39). Research shows that up to 90% of patients in a 100-bed acute gynaecology ward in LRS have pregnancy-related complications requiring PCB (40). The World Health Organization (WHO) now explicitly recommends providing PCB to women seeking gynaecology procedures such as miscarriage treatment and uterus evacuation-related procedures (25).

Designing a medical device intervention for PCB that can be integrated into the healthcare system in LRS is paramount. This ensures that medical devices can match the local conditions and meet the needs of the local people (14, 41, 42). Similarly, it ensures aspects important to low-resource healthcare systems such as affordability, availability, accessibility, appropriateness, and robustness of the device, for example, after multiple use, chemical or steam reprocessing cycles are considered (43, 44). Designing new medical devices for PCB, while considering aspects important to low-resource healthcare systems, can ensure local healthcare facilities no longer have to depend on donations to provide health services.

Recently, new initiatives designing medical devices to be integrated into low-resource healthcare systems have emerged. This is demonstrated in the design of a blood salvage device for ruptured ectopic pregnancy in district hospitals in LRS in SSA (45), uterine balloon tamponade to treat postpartum haemorrhage in under-resourced settings (46, 47), and the design of a portable ultrasound unit for healthcare service in the Lugufu refugee camp, Tanzania (48). Other initiatives further leverage context-driven approaches to consider factors critical to the healthcare system in LRS throughout the medical device design process. This is demonstrated in the context-driven design of an electrosurgical unit (49) or the context-driven design of a retractor for abdominal insufflation-less surgery (50).

**Figure 1:**
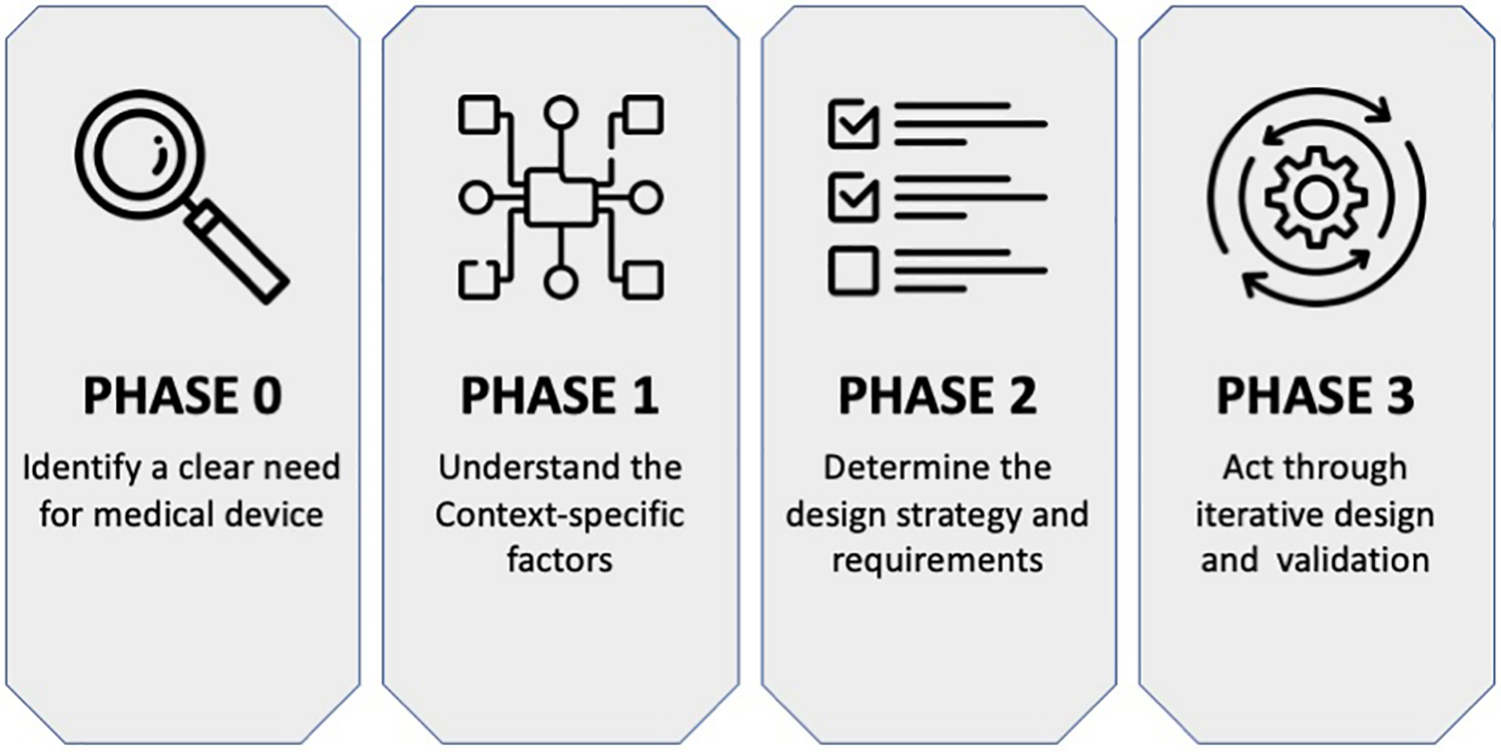
The context-driven design approach for medical devices as proposed by Oosting (51).

The use of a context-driven approach, for example, as proposed by Oosting (51) and summarised in Figure 1, places a necessary emphasis on understanding the nuances particular to designing medical devices for low-resource healthcare contexts. This approach takes the form of: first, identifying a clear need for a new medical device (Phase 0), then exploring context-specific factors such as patient barriers to accessing care within the local healthcare system (Phase 1), followed by developing requirements for the new medical device (Phase 2), and, finally, carrying out device design and validation actions with local stakeholders (Phase 3). This approach can be instrumental in the design of a medical intervention for PCB in LRS. The approach aims to ensure PCB-related contextual factors are identified and understood. Similarly, this approach can guide the development and validation process for achieving a medical device intervention that fits into the low-resource healthcare system and is used over time to provide healthcare to women in need of PCB.

Ensuring that medical devices are designed to fit the local context and used over time in LRS is beneficial for multiple reasons. Firstly, medical devices will be available to support healthcare provision. Secondly, using medical devices over time can curb the reliance on single-use disposable medical devices that contributes to the 282,447 tonnes of waste generated by the healthcare sector in Africa each year (52, 53). Single-use disposables are representative of a linear (or “take-make-waste”) economy in which products are manufactured, used once, and disposed (54). This inherently unsustainable model of production, consumption, and disposal contributes to global environmental destruction (54) and is expected to grow with the increasing global population (55, 56).

**Figure 2:**
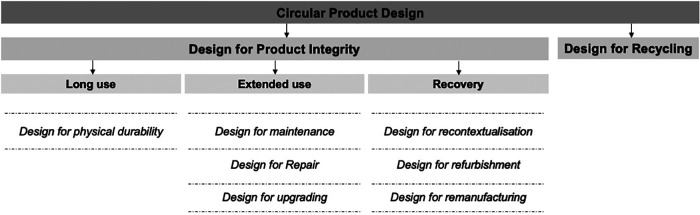
Circular economy design principles as proposed by den Hollander (58).

One of the ways to curb the environmental impact caused by the healthcare sector is to move from medical devices operating in a linear “take-make-waste” economy into a circular economy. In a circular economy, the economic and environmental value of products and their constituent materials is preserved and used for as long as possible (54, 57). This can be achieved by designing durable, maintainable, repairable, upgradable, recontextualised, remanufactured, and recyclable devices, as detailed by den Hollander (58) and summarised in Figure 2. These circular economy principles ensure product, material, and environmental sustainability over time (57) and are therefore essential to be incorporated in the design of medical devices such as the one needed to provide PCB in LRS.

A review of the scientific literature shows there are no documented attempts to design a medical device to provide PCB that meets the context-specific needs of LRS while considering matters of circular economy. For this reason, this research adopts a novel conceptual and practical framing. The research outcome catalogues the development of an actual new medical device that can be used to support the provision of PCB in LRS in SSA—an outcome that aligns with the United Nations’ Sustainable Development Goal 3—Good health and well-being. Furthermore, this study will demonstrate the possibilities and tensions that arise when developing medical devices for LRS while considering context-specific requirements and circularity issues of product, material, and environmental sustainability. In this article, we present the design of a medical device used to support the provision of the PCB in LRS in SSA while leveraging context-driven and circular economy approaches.

## 2. Method

A context-driven design approach was applied to design a medical device to support the provision of PCB during gynaecological procedures in LRS in SSA while leveraging circular economy principles (see Figures 1, 2). The context-driven design approach emphasises understanding the nuances particular to low-resource healthcare contexts for innovating appropriate solutions. At the same time, circular economy principles emphasise product, material, and environmental sustainability needed in the healthcare domain. Below, we describe the implementation of this approach in two parts, that is, Phase 0–2—understanding essential needs and requirements for the medical device design and Phase 3—concept development and validation.

### 2.1. Phase 0–2—understanding essential needs and requirements for design

The starting point for designing a medical device to support PCB during gynaecological procedures in LRS was to understand the context of use (51, 59). This included, for example, the device users (healthcare workers), their needs, tasks involved in administering PCB, why and how these tasks are performed, and barriers encountered by the patients in accessing PCB. To achieve this, extensive field research was conducted in LRS in Kisumu, Kenya, including semi-structured interview discussions in five healthcare facilities. These healthcare facilities were onboarded as partners in this study and included one county (provincial) referral hospital, one primary hospital, and three health centres (one public and two NGO-based), as shown in Table 1. Though PCB and other gynaecological interventions are needed in all sub-Saharan countries, we prioritised Kenya as an entry point due to our vast network with local hospitals, knowledge of local production, and medical device regulatory systems. In the future, we expect to conduct similar research in three other Western, Central, and Southern regions of SSA.

**Table 1:** Healthcare facilities and respective participants interviewed.

Hospital level	Expertise	No. of participants	No. of interviews
County (provincial) referral hospital	Medical doctor—OB/GYN	4	9
	Nurses	3	2
	MO	3	3
Primary hospital (private hospital)	MO	1	1
Public health centre	Nurses	2	2
NGO-based health centre 1	MO	1	1
NGO-based health centre 2	Medical doctor—OB/GYN	1	8
Total		15	26

MO, Medical Officer.

Within these healthcare facilities, 15 participants (as seen in Table 1) were available to be interviewed: five obstetricians/gynaecologists (OB/GYN), five nurses, and five medical officers. The semi-structured interviews were carried out while these healthcare workers performed their respective tasks on PCB or related gynaecological procedures such as the administration of PCB. In some cases, participants were interviewed more than once to gather more information about the PCB and other gynaecological procedures in the local context, as seen in Table 1. Concurrent with the semi-structured interviews, observations were carried out in these healthcare facilities. This included the observation of gynaecological processes such as MVA, which requires the administration of a PCB before uterus evacuation procedures. Likewise, the hospital operating theatre was observed to understand how medical devices for gynaecological procedures are decontaminated, sterilised, and stored before and after use. During interviews and observations, information was recorded as field notes, which were entered into MAXQDA for analysis.

In MAXQDA, data analysis was performed to generate and specify design requirements. The analysis was done by descriptive coding. During descriptive coding, a text fragment was highlighted and assigned a code (60, 61) when information pertaining to a design requirement was mentioned in the interview discussion notes. Codes were derived from the context-driven design approach for surgical equipment (51) and summarised in Table 2. These codes present context-specific factors for establishing design requirements for developing medical devices specifically for LRS.

**Table 2:** Codes and list of requirements for the design of a medical device to support PCB.

Code	Code description as derived from Oosting (51)	Context-specific design requirement	
		Design requirement	Identifier
MD-N	Identify a clear need for certain surgical equipment in a specific context.	Design a medical device that must assist the administration of pain control medication during a paracervical block during gynaecologic procedures. These paracervical block procedures are administered in public and private hospitals in LRS.	M1
CF-B	Identify the different types of healthcare facilities that will be using the medical device needed.		
CF-C	Identify the (surgical) procedures that need to be performed with the medical device needed.		
CF-A	Identify and design against barriers encountered by patients seeking (surgical) care.	The device must be able to reach and provide pain control in the cervix/uterus.	C1
CF-D	Identify the need to provide and/or organise anaesthesia, sterilisation.	The device must be cleaned and sterilised using locally available methods of disinfection and sterilisation. These include high-level disinfection by means of using a chemical solution or the use of pressurised steam or heat sterilisation in an autoclave.	C2
CF-E	Who is part of the team providing surgery, and how are they trained͍?	The device must be easy to use by medical personnel after having undergone training on the device use. Medical personnel include doctors, nurses and midwives, clinical offices, and anaesthetists. These medical personnel are also involved in the procurement (via the procurement department) of the device.	C3
CF-F	Identify who is involved during procurement and usage of device.	Design the device to be locally accessible and available. Health workers should be able to access the device and/or its related accessories locally without relying on import.	C4
CF-G	Is the infrastructure working properly (water, electricity, etc.)?	Ensure the device can function in areas without electrical power grid connection.	C5
CF-H IS-F	Identify what other equipment is available and used. What type of accessories are required (consumables or reusable)	The device must leverage on existing medical devices such as 10-cc syringes locally available. For example, a solution that extends locally available standard 10-cc syringe with standard 18 or 22-gauge needles and provides additional length to administer the paracervical block in the uterus/cervix.	C6
IS-A IS-B	Determine if equipment will be bought, donated, or leased by the hospital͍. What costs are feasible͍?	Ensure the device is affordable, costing (selling price) approximately between US$ 4 and 50. This price range is comparable with the prices of other devices used in procedures requiring a paracervical block. For example, a manual vacuum aspiration kit.	I1
IS-C	What is required to make the device durable?	The device must be reusable multiple times. For example, 25–400 use cycles or more after disinfection and sterilisation.	I2
IS-D	How will maintenance and repair be organised͍?	Design the device such that after-sales services can be provided. This includes providing spare parts for repair, maintenance, and upgrade. Or in other cases, recovery (that is through recontextualisation, refurbishment, and remanufacturing) and/or recycling of obsolete parts. Ensure the device must be manufactured through locally available large-scale or decentralised manufacturing processes that support local after-sale services. Ensure the device can be included and sold together with other existing devices used in procedures requiring a paracervical block. For example, the device could be sold in a pack with 10-cc syringes or sold in a pack with existing MVA kits.	I3 I4 I5
IS-E	Determine how the relationship between the providers of the equipment and the hospital will be during the usage and disposal of equipment͍.		

Using the established codes (see Table 2), the coding exercise was performed. After the first coding round, second and third iterations were conducted. This resulted in a list of coded segments (see Supplementary Data 1) that specify requirements to guide the design of a medical device to support the provision of a PCB. The coded segments were re-written by the design team into actionable design requirements as seen in Table 2. Finally, the design requirements were presented to the healthcare workers in Table 1 for a member-check. Member-check is a technique for exploring the credibility of results (62). Data or results are returned to participants to check for accuracy and resonance with their experiences (62). Each healthcare worker was assigned to read the design requirements (see Table 2) and, after that, remove or provide additional requirements for designing a medical device for PCB specifically for LRS. None of the healthcare workers opposed a design requirement or proposed additional requirements that were not already captured.

### 2.2. Phase 3—concept development and validation

**Figure 3:**
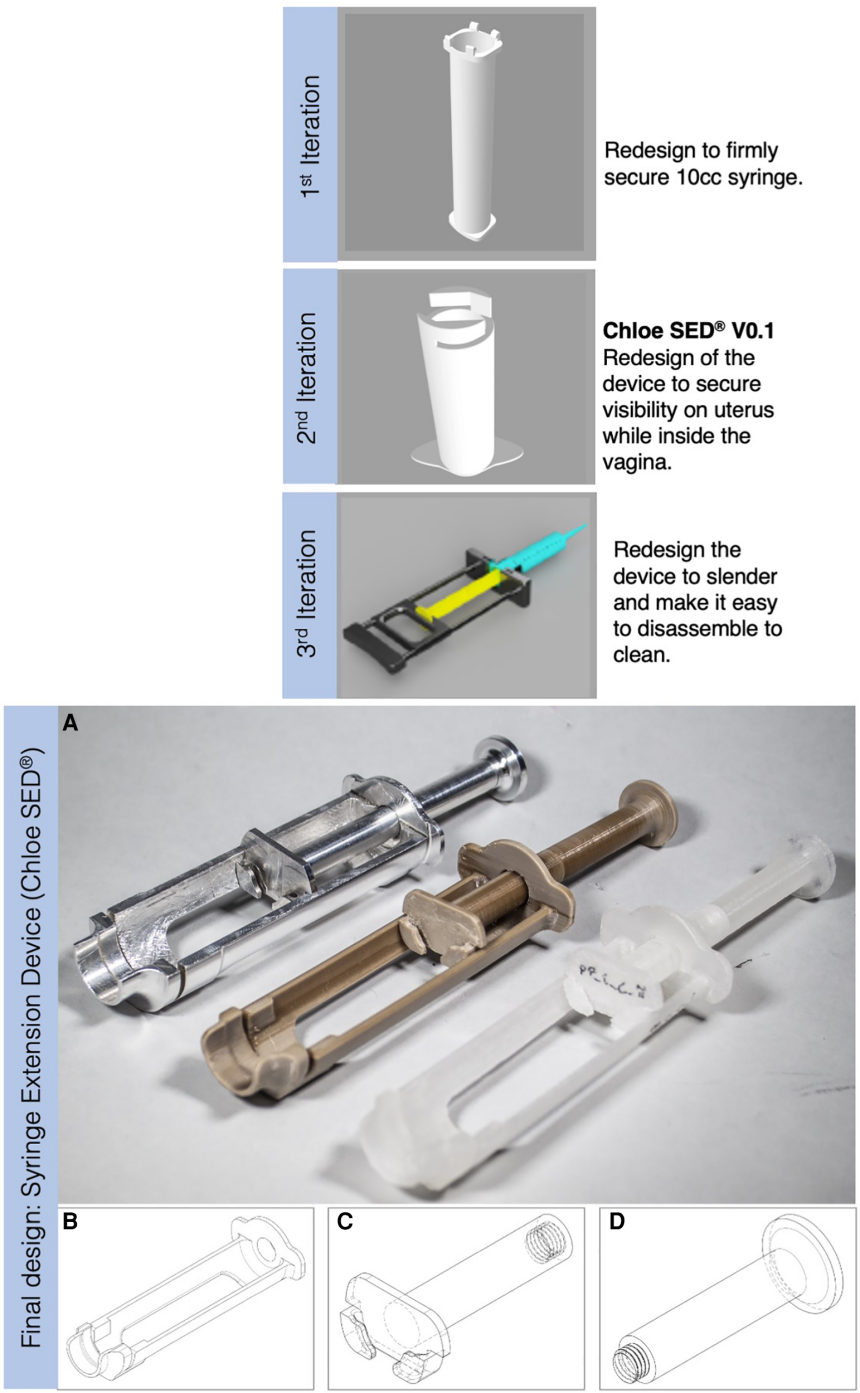
Iterative design process that resulted in a final design of the syringe extension, where (**A**) from left to right is the Chloe SED® manufactured in aluminium grade 6061 and attached with a 10-cc syringe, Chloe SED® in PEEK, and the rest is the device in homopolymer PP with one of them attached to a 10-cc syringe. (**B**) Body, (**C**) plunger, and (**D**) thumb-press are the modular parts of the device.

Phase 3 comprised activities necessary to move from specified design requirements into physical and tangible design artefacts. Using the established design requirements (see Table 2), design ideas and prototypes were developed through a Waterfall Design Process (59, 63). This process allowed for structuring iterative design activities from early conceptual designs through analysis and testing (63) while enabling stakeholders from the five partner healthcare facilities in Kenya to evaluate designs and contribute ideas throughout the process. The activities performed during this concept development stage resulted in a final design called a syringe extension device (Chloe SED®) and manufactured in three specific material options. Figure 3 shows the syringe extension device (Chloe SED®) achieved after three successive design iterations. All concepts were 3D-modelled in Solidworks Education Edition.

Homopolymer polypropylene (PP), polyetheretherketone (PEEK), and aluminium (6061 grade) were selected as the material options for manufacturing Chloe SED®. These materials were selected for several reasons. Firstly, these materials were available for manufacturing in LRS by means of 3D printing or injection moulding manufacturing techniques in the local context. Secondly, these materials have been widely established in science and practice to be safe in manufacturing medical devices intended to be used on patients seeking care (64–66). Also, these materials are durable and can be reused multiple times after reprocessing through high-level chemical disinfection (HLD) and chemical or steam (autoclave) sterilisation (67).

Product design validation activities were conducted at five key design evaluation milestones. These were (1) evaluating whether Chloe SED® fulfilled the established design requirements, (2) evaluating the device's structural quality using finite element analysis, (3) evaluating the extent to which the device is reusable and durable after the expected amount of use cycles, (4) environmental analysis of the device through a life cycle assessment (LCA), and, lastly (5) evaluating the clinical utility of the device when used to administer PCB within a clinical trial. Below, we provide details about each of the evaluations carried out.

#### 2.2.1. Evaluation of final design against the established context-specific design requirement

The first validation step evaluated the extent to which the Chloe SED® met the context-specific design requirements. This evaluation represented an important milestone in the development cycle to assess how effectively the essential needs and design requirements have been captured. The validation activity was designed as a structured questionnaire feedback conducted after using the device to administer a PCB on a life-size female pelvis model. A total of five respondents at a county (provincial) referral hospital conducted this evaluation: two OB/GYN, two nurses (midwives), and a healthcare researcher on interventions for PCB. This evaluation was conducted under ethical clearance NO. PPB/ECCT/21/10/03/2022 (113). Each respondent received an initial briefing about the Chloe SED® and the purpose of the evaluative study. They were then asked to complete two tasks: (1) use the device to perform a simulated administration of PCB on a life-size female pelvis model, and (2) complete a questionnaire that measured the extent to which the final design met the context-specific design requirements. A “Yes” or “No” response on whether the final design met context-specific design requirements C1, C5, and C6 (see Table 2) was sufficient. Measuring the extent to which the final design met design requirements, M1, C2, C3, C4, and I1-I5 (see Table 2) required opinions with a greater degree of nuance than a simple yes or no answer. As such, participants indicated on a Likert scale whether they “strongly agree,” “agree,” “neutral,” “disagree,” or “strongly disagree” that the final design met these requirements.

#### 2.2.2. Finite element analysis to test for the structural quality of the final design

A finite element method analysis in Solidworks Education Edition 2022 was performed to assess the Chloe SED® structural quality. Before analysis was performed, homopolymer PP, PEEK, and aluminium (6061 grade) material properties were applied to the final design model in Solidworks Education Edition 2022. Finite element analysis conditions as set in Solidworks were standard measure mesh type, solid mesh element type, and point load and fixed geometry boundary conditions. This analysis was essential to confirm that the device will not fail, excessively deform, or otherwise be rendered ineffective when impacted with a maximum expected force of 24 N (including a safety factor of 3 as established in Supplementary Data 2). Consequently, von Mises stress and displacement values were measured against force exerted on the device. von Mises stress measures the internal resistance per unit area of a body to an external applied force (68) and the displacement is determined in response to the applied force (69).

#### 2.2.3. Evaluation of the reusability and durability of the device after reprocessing

A reprocessing test was performed in a laboratory setting to evaluate the extent to which Chloe SED® is reusable and durable after expected amounts of use cycles. Reprocessing was performed through HLD or sterilisation. These two reprocessing methods are commonly used in LRS (70–73). It was expected that the device would be reprocessed likewise and thus was evaluated in this study. Only the Chloe SED® manufactured in homopolymer PP and PEEK using 3D printing were reprocessed and evaluated. 3D printing offered an affordable option to manufacture a few samples instead of injecting moulding. However, injection moulding remains a viable option for mass production in future. The device manufactured in aluminium was not reprocessed and evaluated in this study. Research showed that medical devices made from aluminium can be reused over 1,000 times after reprocessing using chemical or steam methods (74) and thus was left out in this reprocessing evaluation study.

The reprocessing of the device through HLD and sterilisation included the following critical steps proposed by the WHO (75). Firstly, decontaminate the device by soaking it in 0.5% chlorine solution for 10 min and rinse it with cool water. Secondly, wash in lukewarm water with detergent, rinse all parts with clean water, and dry by air or with a clean towel. Thirdly, for HLD, soak the device in 2% glutaraldehyde for 20 min and remove with sterile gloves or forceps. Alternatively, for sterilisation, soak in 2% glutaraldehyde for 10 h and remove with sterile gloves or forceps. Lastly, rinse under running sterile water, then air dry or dry with a sterile cloth, and reuse the device.

Following the established reprocessing procedure, four Chloe SED® prototypes (two in PP and two in PEEK) and 20 3D printed standard American Society for Testing and Materials (ASTM) dog bones samples (10 in PP and 10 in PEEK) were reprocessed. Reprocessing the prototypes was explicitly aimed at evaluating the extent to which Chloe SED® is reusable after reprocessing. The reprocessing of the standard ASTM dog bones was aimed at evaluating the device's durability in terms of tensile strength after reprocessing. In evaluating the extent to which the device is reusable after reprocessing, one Chloe SED® prototype in PP and another in PEEK were subjected to reprocessing using HLD. Similarly, one prototype in PP and one in PEEK were subjected to reprocessing through chemical sterilisation. After every reprocessing cycle, each prototype was assembled, used, and examined for any damages. Damages examined included cracks, breakages, or part shrinkage that would render the device unusable. A total of 25 reprocessing cycles were performed and examined for any damages. Twenty-five cycles were performed since similar devices used for gynaecological procedures requiring PCB in LRS, such as IPAS MVA kit, are reprocessed through HLD or sterilisation up to 25 times (76). As such was a suitable base for comparison.

Lastly, in evaluating the extent to which the Chloe SED® remains durable after reprocessing, five standard ASTM dog bones in PP and five in PEEK were subjected to reprocessing using HLD. Similarly, five standard ASTM dog bones in PP and five in PEEK were subjected to reprocessing through chemical sterilisation. After 25 reprocessing cycles, a tensile test was performed on each of the reprocessing ASTM dog bones to measure the durability in terms of tensile yield strength.

#### 2.2.4. Life cycle assessment and environmental impact

To assess the environmental impact of the production and (re)use cycles of the Chloe SED®, an LCA (77) was performed. LCA looks at the environmental impacts and resources used throughout a product's life cycle, from raw material acquisition, via production and use phases, to the end-of-life (78). This was performed using Activity Browser software, which builds on the brightway2 python package for LCA calculations. The main data source was Ecoinvent v3.9.1, augmented with literature values for PEEK from Hytechcycling RefA 08/05/2018 (79). This study's LCA assessed the environmental impacts of material sourcing (homopolymer PP, PEEK, and aluminium 6061 grade) and production of the final design using injection moulding technique, and its use phases in the healthcare facility.

Because the exact intended manufacturing materials (homopolymer PP, PEEK, and aluminium 6061 grade) or production process (injection moulding) were unavailable in the Ecoinvent v3.9.1 database, proxy materials and processes were used. Granulated PP and wrought aluminium alloy were used as alternatives to homopolymer PP and aluminium 6061 grade, respectively. Pipe and section bar extrusion was used as alternation production techniques for injection moulding the final design in PP and aluminium, respectively. End-of-life was not taken into account due to the complexity of defining the exact end-of-life pathways in the local context within the timeframe of this research study.

The sterilisation process in Ecoinvent v3.9.1 was modelled as follows. A single sliding door horizontal autoclave with an energy consumption of 7 kWh per cleaning cycle (1.5–2 h per cycle) of 40 kg of material was considered. Note that a single sliding door horizontal autoclave was available at the County (provincial) referral hospital as in Table 1 above. 6% sodium hypochlorite was used to model chemical HLD or sterilisation since 2% glutaraldehyde was unavailable in Ecoinvent v3.9.1. Both chemicals are universally accepted for medical device sterilisation (80, 81). Data on chlorine solution and water, which are needed for chemical HLD or sterilisation, could be inferred in Ecoinvent v3.9.1.

Before the LCA was performed, a quantified description (also known as a functional unit) that serves as the reference basis for all calculations regarding impact assessment was established. The quantified description (functional unit) was defined as the “use of final device design in a hospital for 1 year.” The number of procedures requiring using the final design within 1 year of clinical operation was approximately 500. This number was established during the semi-structured interviews within the healthcare facilities detailed in Table 1. During these interviews, it was noticed that each healthcare facility performed approximately three to nine procedures requiring PCB weekly. Taking an upper limit of nine procedures per week amounts to 477 procedures per year. For easy calculations, the number of procedures per year was rounded up to 500.

Note that the number of reuse cycles of a medical device varies as per the reprocessing technique and thus affects the number of devices needed as per the functional unit. For example, a medical device in PP can be reprocessed using chemical sterilisation for approximately 25 terms (82–84) and in an autoclave approximately five times (85, 86) before deformation. In effect, this means the number of Chloe SED® needed as per the functional unit will be 100 or 20 when prioritising reprocessing in an autoclave or chemical sterilisation, respectively. Table 3 shows the number of devices needed per LCA functional unit when prioritising chemical or autoclave sterilisation. Based on these established parameters, the LCA was performed to measure the environmental impact of the syringe extension device as per the defined functional unit. Detailed calculation of the LCA can be seen in Supplementary Data 3. The selected impact category was Intergovernmental Panel on Climate Change (IPCC) 2021 GWP100, so that the unit of analysis is kg CO_2_-eq.

**Table 3:** Number of devices needed as per the LCA functional unit.

Chloe SED® device type (in material)	Device weight (grams)	Reprocessing method and reuse cycles		Number of devices needed per year as per the functional unit
		Method	Estimated reuse cycles as per material type	
Homopolymer PP	151	Autoclaving	5 (85–86)	100
		Chemical sterilisation	25 (82–84)	20
PEEK	220	Autoclaving	25	20
		Chemical sterilisation		
Aluminium (6061 grade)	451	Autoclaving	1,000 (74)	0.5
		Chemical sterilisation		

#### 2.2.5. Clinical trial

The last validation test in the form of a clinical trial under the approval of the Poison and Pharmacy Board of Kenya—NO. PPB/ECCT/21/10/03/2022 (113) was performed. The clinical trial evaluated the clinical utility and effectiveness of the device in providing a PCB to patients in need. This clinical study is ongoing, and results will be communicated in a follow-up publication.

## 3. Results

### 3.1. Concept development and validation

**Figure 4:**
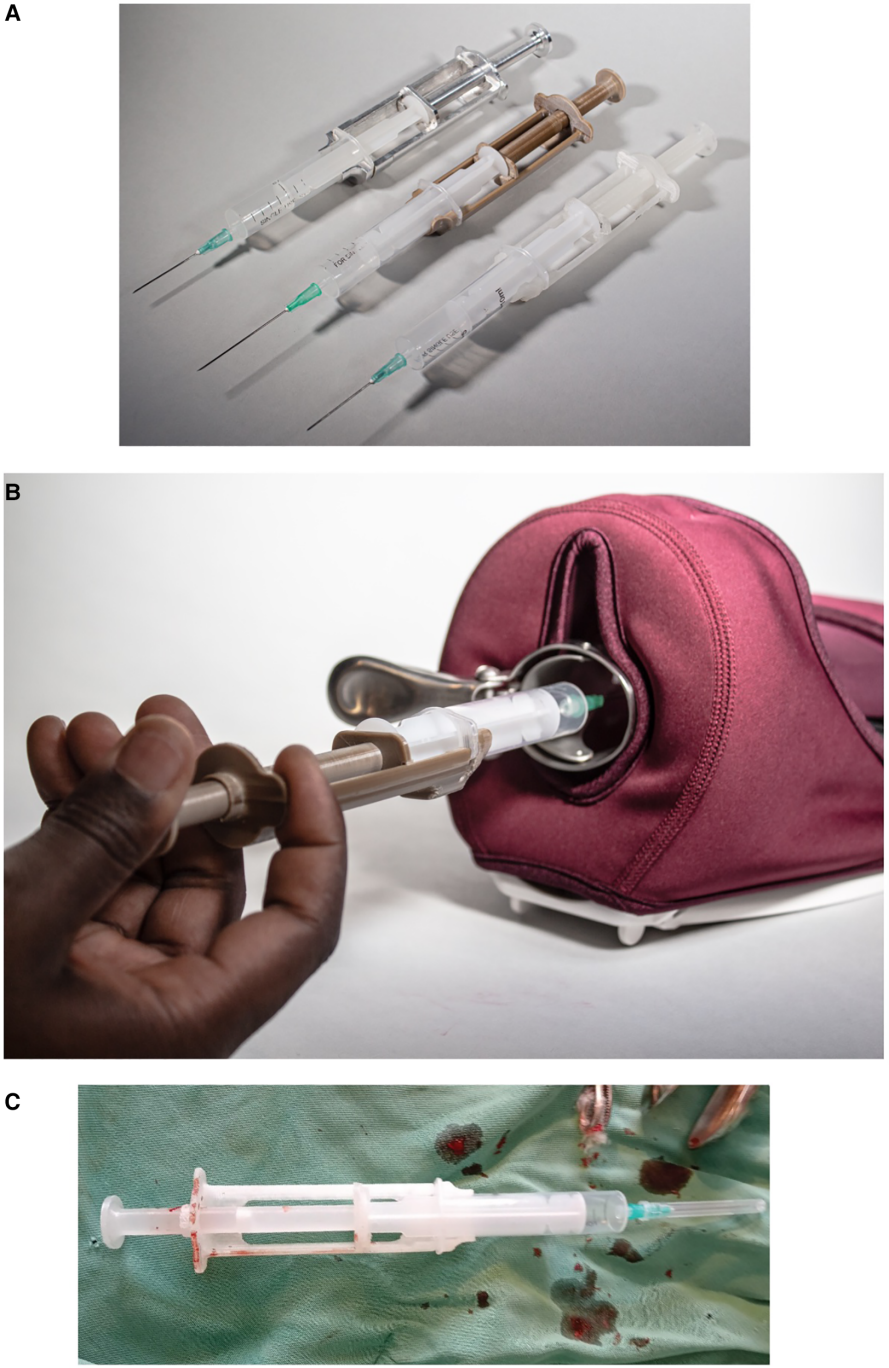
Syringe extension device (Chloe SED®) where (**A**) is the different Chloe SED® models attached to a 10-cc syringe; (**B**) is a hand size demonstrator of Chloe SED® on a pelvic model; and (**C**) Chloe SED® used in the local context to provide paracervical blocks.

The outcome of the context-specific design of a medical device to support the provision of a PCB while leveraging on circular economy principles resulted in a syringe extension device (Chloe SED®). This device is snap-fitted on any 10-cc syringe to provide additional length to reach the cervix during PCB and other gynaecological procedures requiring a syringe extension (see Figure 4). Supplementary Data 4 shows an example of 15 different 10-cc syringes collected from healthcare facilities in the context and used on the device.

Chloe SED® was designed in three modular parts, that is, body, plunger, and thumb-press (see Figures 3B–D). The modular design provided the opportunity to maintain, repair, and upgrade the individual parts when needed without affecting the other parts. For example, repairs can be made by replacing a malfunctioning thumb-press instead of disposing of the device as a whole. Upgrades can be achieved by offering Chloe SED® body and plunger parts for syringes smaller than 10-cc without changing the thumb-press. Similarly, these modular parts can be recovered for recycling when the device or its constituent parts break and reach their end of life. The ability to repair, maintain, upgrade, and recycle this device were the circular economy principles successfully integrated within this study. Circular economy principles such as refurbishment or remanufacturing were not integrated into the design of the Chloe SED®. Incorporating these principles in the Chloe SED® required industrial processes that were technically or financially not feasible for local manufacturers. The device is estimated to cost (selling price) US$ 1.5 per device when produced in homopolymer PP, US$ 10 per device in PEEK, and US$ 15 in aluminium grade 6061. These are estimated prices per injection moulding of the device with a minimum production of 1,000 units locally in Kenya.

#### 3.1.1. Evaluation of Chloe SED® against context-specific design requirements

Seeking to evaluate the proposed design, healthcare workers rated the extent to which the Chloe SED® met the established context-specific factors in Table 2. All the healthcare workers responded with a “Yes,” indicating that the Chloe SED® device met the design requirements C1, C5, and C6 (Table 2 details these requirements). These design requirements concerned the ability of Chloe SED® to reach and provide pain control in the cervix, function in areas without electrical power grid connection, and leverage on locally available 10-cc syringes, respectively. Similarly, all the ratings except for two either “strongly agreed” or “agreed” that the design met the rest of the other context-specific requirements as seen in Table 4. Two context-specific factors were rated “neutral,” that is, design requirements I1 and I3 (see Table 4). These factors concerned the affordability of the device and the after-sales services in providing spare parts for repair, maintenance, upgrade recovery, and recycling of obsolete parts. These ratings were marked neutral since the participants doubted whether providing after-sales services would influence the initial or operating cost of the device.

**Table 4:** Ratings that show the extent to which the final designed Chloe SED® fulfilled the established context-specific design requirements.

Design requirement		Strongly disagree	Disagree	Neutral	Agree	Strongly agree
Identifier	Requirement					
M1	Chloe SED® is designed to assist in the administration of pain control medication during paracervical blocks during gynaecologic procedures.	0%	0%	0%	20%	80%
C2	Chloe SED® can be cleaned and sterilised using locally available methods of disinfection and sterilisation. These include high-level disinfection by means of using a chemical solution or the use of pressurised steam or heat sterilisation in an autoclave.	0%	0%	0%	60%	40%
C3	Chloe SED® is easy to use by medical personnel after having undergone training on the device use. Medical personnel include doctors, nurses and midwives, clinical officer, and anaesthetics. These medical personnel are also involved in the procurement (via the procurement department) of the device.	0%	0%	0%	40%	60%
C4	Chloe SED® is designed to be locally accessible and available. Health workers can be able to access the device and/or its related accessories locally without relying on import.	0%	0%	0%	60%	40%
I1	Chloe SED® is affordable, costing (selling price) approximately between US$ 4 and 50. This price range is comparable with the prices of other devices used in procedures requiring a paracervical block. For example, a manual vacuum aspiration kit.	0%	0%	20%	40%	40%
I2	Chloe SED® is designed to be reusable multiple times. For example, 25–400 use cycles or more after disinfection and sterilisation.	0%	0%	0%	60%	40%
I3	Chloe SED® is designed such that after-sales services can be provided. This includes providing spare parts for repair, maintenance, and upgrade. Or in other cases, the recovery (that is through recontextualisation, refurbishment, and remanufacturing) and/or recycling of obsolete parts.	0%	0%	40%	20%	40%
I4	Chloe SED® can be manufactured through locally available large-scale or decentralised manufacturing processes that support local after-sale services.	0%	0%	0%	80%	20%
I5	Chloe SED® can be included and sold together with other existing devices used in procedures requiring a paracervical block. For example, the device could be sold in a pack with 10-cc syringes or sold in a pack with existing MVA kits.	0%	0%	0%	20%	80%

#### 3.1.2. Finite element analysis to test t Chloe SED® structural quality

**Figure 5:**
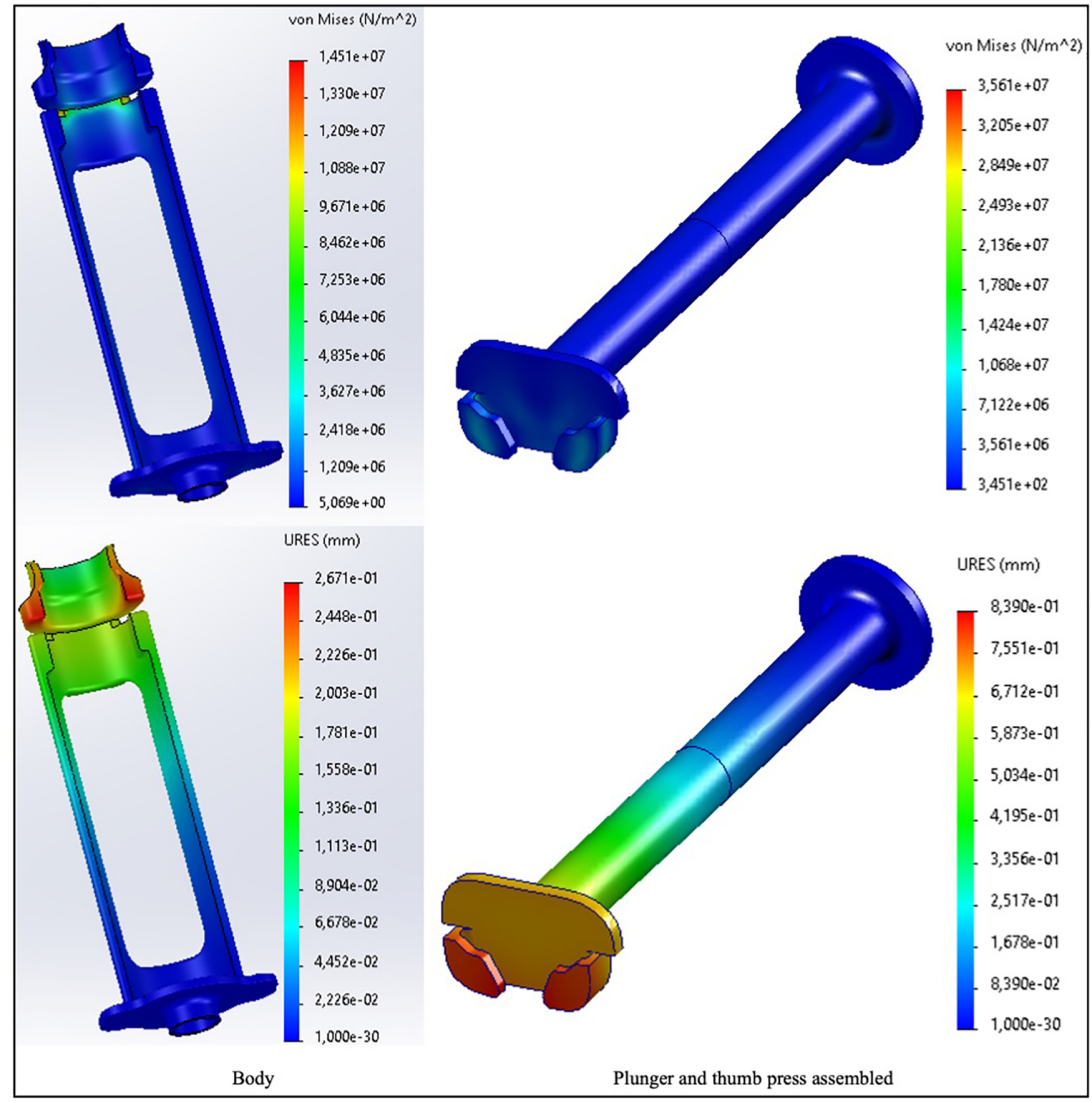
Finite element analysis simulation in von Mises stresses and resulting displacement (URES) for the Chloe SED® (body and plunger–thumb-press assembly) in PP.

Finite element analysis performed to check for the structural quality of Chloe SED® resulted in the following. The analysis showed that when impacted with a maximum expected force of 24 N, the Chloe SED® presented stress-displacement levels. Figure 5 highlights an example of the finite element analysis outcome for the device in PP and the most likely failure points by displaying the regions of lowest stress-displacement levels (in blue) to greatest (in red). Stress levels between 14.5 and 35.6 MPa and displacement of 0.27–0.84 mm were presented for Chloe SED® in PP (as illustrated in Figure 5), 13.1–27.4 MPa and displacement of 0.12–0.38 mm for PEEK, and 13.3–29.5 MPa and displacement of 0.007–0.02 mm for aluminium. See Supplementary Data 5 for the simulations of PEEK and aluminium.

The stress levels of Chloe SED® in PEEK and aluminium are acceptable since they are lower than the material yield strength, that is, 70–103 MPa for PEEK and 124–290 MPa for aluminium. These lower stress-displacement levels imply minimal deformation, and the device's efficacy will be unaffected by the required force needed to operate the device. On the other hand, the stress levels of Chloe SED® in PP (14.5–35.6 MPa) fall within the yield material strength of 19–45 MPa and thus increase the chances of PP failing.

#### 3.1.3. Evaluation of the reusability and durability of Chloe SED® after reprocessing

**Figure 6:**
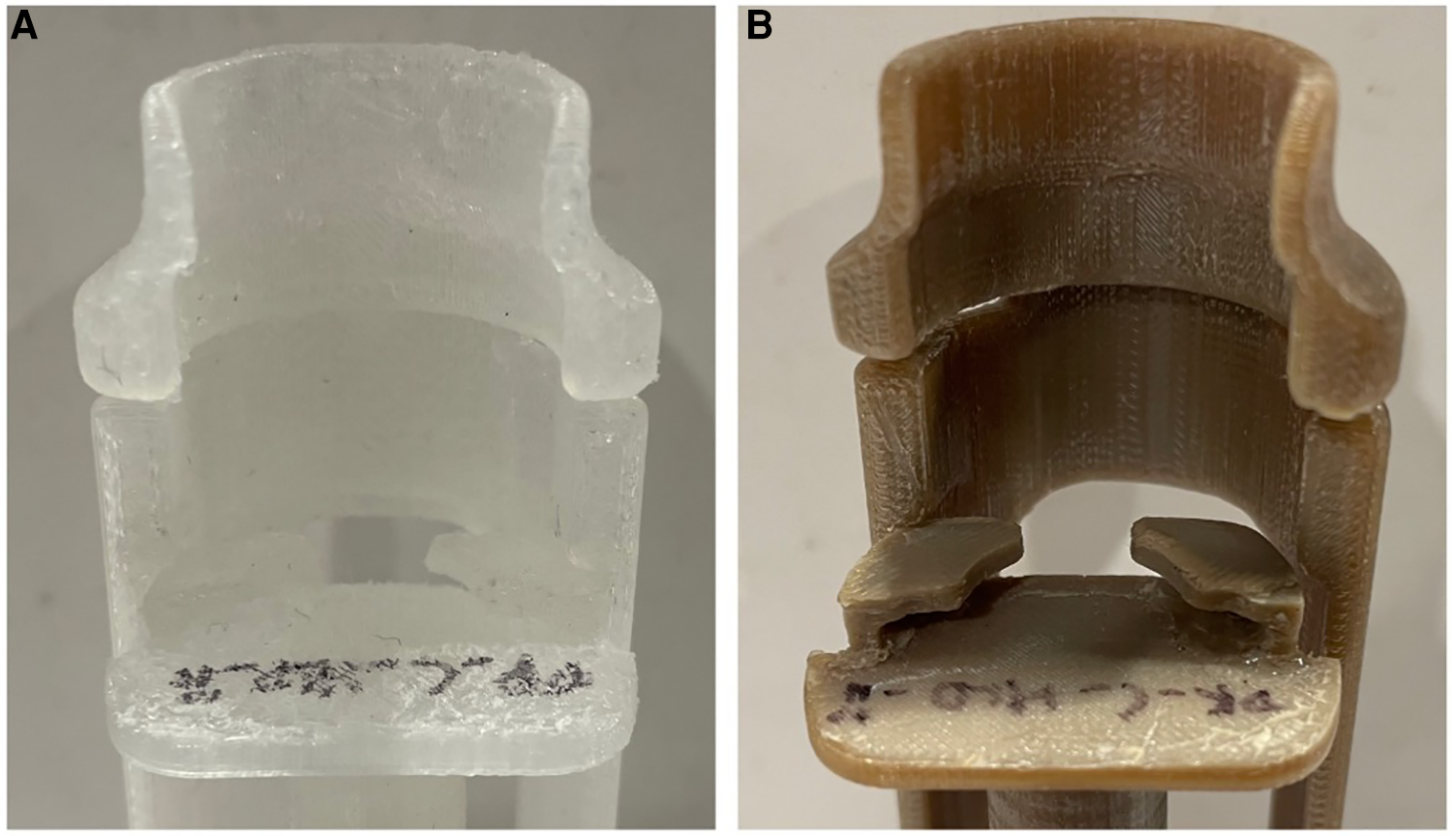
Image showing slight surface wear after 25 cycles of reprocessing (**A**) Chloe SED® in PP and (**B**) in PEEK.

The evaluation to check for the reusability of the Chloe SED® after repeated use cycles and reprocessing using HLD or sterilisation resulted in the following. All four Chloe SEDs® (two in PP and two in PEEK) were still in good condition and reusable after the 25 cycles. The devices were functional as a 10-cc syringe could be firmly attached and used to pull in and push out liquid through the syringe needle. Similarly, none of the devices was observed to be broken or had any cracks. On the other hand, slight surface wear on the four reprocessed Chloe SEDs® was noticed, as seen in Figure 6. The shrinkages and surface wear were more noticeable in the Chloe SED® printed in PP material than on the Chloe SED® in PEEK.

Similarly, the tensile strength test of 20 dog bone samples (10 in PP and 10 in PEEK) aimed at evaluating the device's durability after multiple reprocessing cycles yielded results as follows. PP samples after 25 cycles of reprocessing had a tensile yield strength ranging from 16.93 to 19.55 MPa while PEEK produced yield strength ranging from 75.15 to 90.27 MPa. The tensile yield strength values fall within the material yield strength. These results imply that PP and PEEK materials used to manufacture the syringe extension device have an increased chance of failing after 25 cycles of reprocessing using high-level chemical disinfection or sterilisation.

#### 3.1.4. Environmental assessment

**Figure 7:**
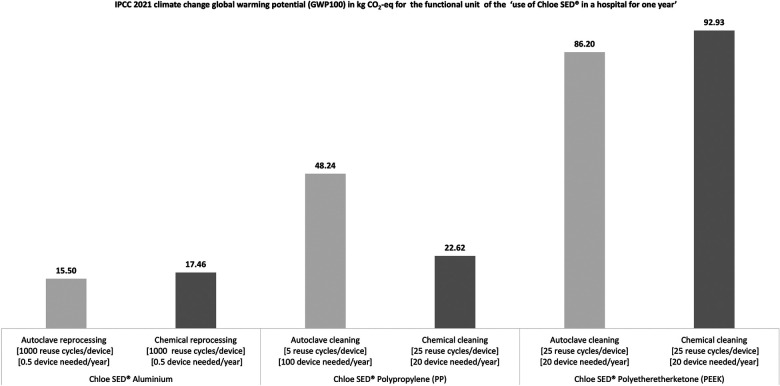
Environmental impact in kg CO_2_-equivalent for the production and use of Chloe SED® in a hospital for 1 year.

The environmental assessment through an LCA as per the functional unit showed that the Chloe SED® in aluminium generated the least environmental impact, regardless of the sterilisation method used as seen in Figure 7. These results imply that the environmental impact of Chloe SED® depends on what type of cleaning method is employed to render the device reusable.

Similarly, the cleaning method employed to render the device reusable affects the number of devices needed to run clinical operations for 1 year in a hospital as per the LCA functional unit (see Figure 7). For example, only 0.5 Chloe SED® in aluminium was needed to run a PCB clinical operation for 1 year. This is related to the fact that aluminium material can be reused over 1,000 times after reprocessing through chemical or steam sterilisation. Similarly, 20 Chloe SED® in PP were needed each year in a hospital if chemical sterilisation is prioritised over autoclaving requiring 100 devices. This can be attributed to the fact that PP as a material has lower reuse cycles (five reuse cycles) when continuously exposed to high temperatures in an autoclave than when exposed to chemical reprocessing (at least 25 reuse cycles). Chloe SED® in PEEK, requiring 20 devices per year, produced the highest environmental impact despite having at least 25 reuse cycles after chemical or autoclave reprocessing. This can be attributed to the fact that PEEK is labelled as an emerging critical material, and the environmental impact of sourcing the raw material and production is much higher than for PP or aluminium. The environmental impact of material sourcing and production of PP, PEEK, and aluminium is 2.3, 17.4, and 13.8 kg CO_2_-equivalent, respectively (see Supplementary Data 3).

## 4. Discussion

This paper presented the design and validation of a medical device to support the provision of PCB during gynaecological procedures in LRS in SSA countries, in this instance, Kenya. The aim of this study was twofold: firstly, to develop a medical device that can be available, affordable, and support healthcare in LRS to provide PCB; secondly, to design the medical device from a context-specific and circular economy perspective. Context-specific approach captured nuances particular to low-resource healthcare contexts, and circular economy accounted for product, material, and environmental sustainability. The design of the medical device started with first understanding the use context, which resulted in a list of context-specific design requirements, next through concept development, and validation while leveraging on circular economy principles. The study's outcome produced a medical device used to support the provision of PCB called the syringe extension device (Chloe SED®).

Chloe SED® is snap-fitted onto any standard 10-cc syringe found in LRS to provide the additional length needed to reach and administer local pain medication around the cervix during PCB or other gynaecological procedures. With this device, healthcare facilities do not have to rely on spinal needles, usually expensive or unavailable in the local context. Instead, healthcare facilities in LRS can make use of the widely available and affordable standard 10-cc syringes attached to the Chloe SED® and administer PCB. Consequently, this study has resulted in the availability of a medical device needed to support women to access PCB, which is vital in sexual reproductive healthcare services such as the treatment of miscarriages and abortions. This is in line with the WHO’s call to support and strengthen the access and availability of medical devices (42), which can be used to provide comprehensive sexual reproductive healthcare access for women in LRS in SSA (25, 87). With the SED costing approximately US$ 1.5 in homogenous PP, US$ 10 per device in PEEK, and US$ 15 in aluminium, healthcare facilities can afford to provide women with PCB services.

For as low as US$ 1.5, healthcare facilities can secure a Chloe SED®, which is reusable between five and 25 times, depending on method of sterilisation. This price point tackles the issue of medical device affordability in LRS. Research shows that an affordable initial cost price is particularly important in LRS due to constraints in financial capacity (88, 89). Healthcare facilities in LRS will likely purchase a medical device at a price below or within their set budget (90, 91). Chloe SED® (at least in PP) costing US$ 1.5 and attached to a 10-cc syringe which costs US$ 0.1 provides healthcare facilities with access to an alternative device for PCB at an initial cost comparable to the currently used disposable spinal needle, which is used only once and is often unavailable or expensive at a price range of US$ 1.5–28. Providing affordable medical devices resonates with agendas of the Sixtieth World Health Assembly in May 2007, which aims at ensuring that medical devices are affordable to populations in need (92).

On the other hand, the initial cost of Chloe SED® in PEEK (US$ 10) and aluminium (US$ 15) is higher than the initial cost of Chloe SED® in PP (US$ 1.5). Though these costs are higher, it, however, provides healthcare facilities with the option to have access to the device with many reuse cycles. As seen in this study (see Section 2.2.3), the SED in PEEK has a reuse cycle of at least 25 times. Estimates in the literature suggest that PEEK materials can be reused more than 25 times (93, 94) and aluminium more than 1,000 times after reprocessing (74). We speculate that the Chloe SED® in PEEK and aluminium will have similar reuse cycles and can be used to support healthcare facilities providing PCB, especially in remote settings where access to spinal needles is difficult or impossible. Suppose affordability issues arise in remote healthcare facilities, the device in PP costing US$ 1.5 remains an option, though having a lower reuse cycle than the device in PEEK and aluminium.

Ensuring many reuse cycles in the design of the Chloe SED® remained a key component, especially when considering aspects of circular economy. In a circular economy, products should remain used in the economic system for as long as possible and thus ensure product, material, and environmental sustainability (95). This study takes into account these circular economy aspects in different ways. Firstly, by designing a durable device through leveraging durable materials (PP, PEEK, aluminium) with multiple reuse cycles. Secondly, the Chloe SED® modular design (see Figure 3) provides the opportunity for maintenance, repair, and upgrade of individual parts without affecting the other parts. As such, the device can remain reused over an extenuating period of time. Thirdly, if the Chloe SED® breaks down beyond repair, its material can be recovered through recycling. Chloe SED® is produced from a single material without colourants and coatings, which makes it suitable for relatively high-quality mechanical recycling. All these factors go a long way to ensuring that the Chloe SED® and its constituents remain in the economic system. However, the environmental impact of this product remains a critical consideration in a circular economy.

The Chloe SED® in PP, PEEK, or aluminium has different environmental impacts regarding CO_2_ emissions. Environmental impact in terms of CO_2_ emissions is a contributing factor to challenging problems of global environmental destruction (96). Considering the manufacturing and use of products with low carbon emission levels is vital in addressing global environmental issues (97), as demonstrated in this study. As seen in Figure 7, Chloe SED® produced in aluminium and reused after reprocessing in an autoclave generated the least environmental impact compared to Chloe SED® in aluminium reuse after reprocessing through chemical cleaning and Chloe SED® in PP produced and reused after reprocessing through autoclaving or chemical sterilisation. This is attributed to the fact that Chloe SED® in aluminium material is durable and has a much higher reuse cycle (1,000 reuse cycle) after reprocessing by chemical or autoclave sterilisation than Chloe SED® PEEK and PP. Based on these facts, the Chloe SED® produced in aluminium and reused over time after reprocessing in an autoclave is more environmentally friendly than the Chloe SED® in PP and PEEK.

A Chloe SED® produced in aluminium with over 1,000 reuse cycles achieved a more environmentally friendly status than that in PP and PEEK with 25 reuse cycles. This means that Chloe SED® in aluminium can be considered a desirable product when prioritising environmental issues of material sourcing and production, and clinical use of the device over time. On the other hand, Chloe SED® in aluminium might be less desirable compared to Chloe SED® in PP when considering factors of the initial device cost often emphasised in healthcare facilities in LRS. In addition, the issue of cost is magnified when considering the initial and operational cost of device reprocessing. For example, the initial cost of an affordable autoclave designed for LRS can be approximately US$ 85–620 or more (98, 99) and possess an operational cost of US$ 50 per hour (100). Similarly, estimates suggest that one cycle of chemical sterilisation can cost US$ 5–10 (101). In essence, healthcare facilities will incur reprocessing costs in order to render any of the Chloe SED® reusable. As such, a correlation between the material choice used to manufacture the device, the device's initial cost, product durability or reuse cycle, reprocessing method and cost, and environmental impact emerge. This correlation is in line with other studies (102) that describe these correlations as conflicting or divergent trade-offs. These trade-offs are interconnected and can include many other societal challenges. Levänen et al. also noted that these trade-offs could be particularly large in LRS and thus go as far as affecting strategic planning or use of a product (103). These trade-offs are inevitable and must be continually assessed to deliver a workable product that achieves the greatest synergy between meeting the needs of people and preserving the environment.

The limitation of our investigation is that it only shows the correlation between material choice, initial cost, product durability, and environmental impact specific to design and production before use. Other aspects such as environmental impact or cost associated with the provision of after-sales services such as repair or maintenance will amplify the conflicting or divergent trade-offs in designing medical devices for LRS. The evaluation of the Chloe SED® against context-specific design, as in Table 4, already starts to demonstrate this trade-off. In Table 4, neutral ratings were provided since the survey participants were conflicted about how providing after-sales services such as repair and manufacturing that can ensure the device remain used in a circular economy would influence the initial or operating cost. Such trade-offs are bound to happen, especially when designing for LRS that are already plagued with resource scarcity, institutional voids, and market affordability (104–106). However, continuous efforts to understand the local context are vital to navigating such trade-offs and delivering functional products that empower local communities (105).

The context-specific design approach used in this study was vital in understanding the local setting and delivering a functional product such as the SED. This approach was necessarily unique as it provided stepwise guidance to understanding the local setting and the medical device design needs. However, in using this approach, it was remarkable to observe that circularity considerations needed to be explicitly detailed. It is vital to explicitly include circularity issues in the design of medical devices. Transformation of the medical device industry to a more circular economy would advance the goal of providing healthcare while considering product, material, and environmental sustainability (54). As such, there is an opportunity for future research to develop context-specific design approaches or tools while ensuring product, material, and environmental sustainability. Such tools can facilitate medical devices to depart from linear operational models into circular ones.

## 5. Conclusion

This study attempted to design a medical device to provide PCB that meets the context-specific needs of LRS in SSA while considering matters of circular economy. Through understanding the context-specific needs in a low-resource healthcare setting and iterative concept development and validation phases, this study catalogues the development of an actual new medical device for PCB called the syringe extension device (Chloe SED®). Chloe SED®, priced at US$ 1.5 per device when produced in homopolymer PP, US$ 10 in PEEK, and US$ 15 in aluminium, is snap-fitted on any 10-cc syringe in LRS to provide PCB for women in need. With this device, low-resource healthcare systems do not have to rely on expensive or often unavailable tools such as spinal needles to provide PCB. By simply attaching a 10-cc syringe to Chloe SED®, healthcare facilities can provide women with PCB required in many gynaecological procedures such as LEEP or treatment of miscarriage or incomplete abortion.

Designing Chloe SED® to be embedded within the healthcare system in LRS was achieved by leveraging on a context-specific design approach. This approach emphasised understanding the nuances particular to low-resource healthcare contexts, such as the availability and affordability of devices that can be used over time to provide healthcare for all. Ensuring that Chloe SED® remained used over time was achieved by leveraging circular economy design principles of durability, repairability, maintainability, upgradeability, and recyclability. These principles ensured that modular parts of Chloe SED® and its constituent material could remain reused for as long as possible in the economic system, thus, desirable from an environmental sustainability perspective.

However, in ensuring that Chloe SED® is desirable for the environment and meets context-specific needs in LRS, correlations between material choice used to manufacture the device, the device's initial cost, product durability or reuse cycle, reprocessing method and cost, and environmental impact emerged. These correlations can be seen as interconnected conflicting or divergent trade-offs and can include many other societal challenges. These trade-offs are inevitable. It is recommended that (biomedical) engineers and medical device designers must continually assess and navigate these trade-offs to deliver a workable product that achieves the greatest synergy between meeting the needs of people and preserving the environment.

Achieving the synergy between meeting the needs of people and preserving the environment in medical device design can be actualised by leveraging on context-specific and circular economy approaches. However, these approaches still operate in a silo. We recommend that designers and researchers can explore developing context-specific design approaches or tools that explicitly consider circularity product, material, and environmental sustainability. Such tools can facilitate medical devices to depart from linear operational models into circular ones.

This study is intended to be seen as an effort to make available medical devices to support women in accessing sexual reproductive health services, specifically in LRS in SSA. With Chloe SED®, healthcare facilities and organisations can continue supporting women with PCB during gynaecological procedures.

## Data Availability

The original contributions presented in the study are included in the article/Supplementary Material, further inquiries can be directed to the corresponding author.
