# Supplementary material for: Design of a syringe extension device (Chloe SED®) for low-resource settings in sub-Saharan Africa: a circular economy approach
Source: Front Med Technol. 2023 Sep 1;5:1183179. doi: 10.3389/fmedt.2023.1183179 (PMC10505716; doi:10.3389/fmedt.2023.1183179)
Supplement: Supplementary file 1 [file Table1.docx]

Supplementary Material

**Design of a syringe extension device (Chloe SED^®^) for low-resource settings in Sub-Saharan Africa. A circular economy approach.**

Karlheinz Tondo Samenjo^1,3*^, Aparna Ramanathan^2,3^, Stephen Gwer^3,4^, Frederick Otieno^3^, Robert Bailey^5,3^, Erin Koksal^6^, Benjamin Sprecher^1^, Rebecca Price^1^, Conny Bakker^1^, Jan-Carel Diehl^1^.

^1^Department of Sustainable Design Engineering, Faculty of Industrial Design Engineering, Delft University of Technology, Delft, Netherlands.

^2^National Center for Advanced Pelvic Surgery, Medstar Washington Hospital Center, Georgetown University, Washington, District of Columbia, USA.

^3^Nyanza Reproductive Health Society, Kisumu, Kenya.

^4^Department of Obstetrics and Gynaecology, Maseno University, Kisumu, Kenya.

^5^University of Illinois at Chicago, School of Public Health, Chicago, Illinois, USA.

^6^Rethink Robotics, Boston, MA.

*** Correspondence:**Corresponding Author
K.T.Samenjo@tudelft.nl

# Supplementary Data 1 - Summaries with Coded Segments

| **Code** | **Coded segments** | **Summary** |  |
| --- | --- | --- | --- |
|  |  | **Requirment** | **ID** |
| MD-N: Identify what medical equipment needed in the context. | extend the length of a standard syringe and needle by about 3-4 inches so that health workers can use the materials available to perform a paracervical anesthetic block (you need the additional length to be able to reach the cervix).  S002 - All interviews, discussions and observations\1Interview S1_7_1MD: 4 - 4 (0) | Design a medical device that must assist the administration of pain control medication during paracervical block during gynecologic procedures. These paracervical block procedures are administered in public and private hospitals in LRS. | M1 |
|  | device is designed to assist in the administration of paracervical block for pain control during gynecologic procedures  S002 - All interviews, discussions and observations\2InterviewS_8_1MD: 3 - 3 (0) |  |  |
|  | effective and safe in providing pain control for women during manual vacuum aspiration  S002 - All interviews, discussions and observations\4InterviewJ_9_2_2MD: 10 - 10 (0) |  |  |
| CF-B: Identify what type of healthcare facilities are target | extend the length of a standard syringe and needle by about 3-4 inches so that health workers can use the materials available to perform a paracervical anesthetic block  S002 - All interviews, discussions and observations\1Interview S1_7_1MD: 4 - 4 (0)  I think they're a little too expensive to work on the community health worker level here: http://www.medicalresources.com/shopping  S002 - All interviews, discussions and observations\1Interview S1_7_1MD: 5 - 5 (0) |  |  |
|  | device is intended for use in designated procedure areas in medical facilities including hospitals and clinics  S002 - All interviews, discussions and observations\2InterviewS_8_1MD: 7 - 7 (0) |  |  |
|  | - Private hospital - General anesthesia - Public - Not always anesthesia, patient buys consumables - S002 - All interviews, discussions and observations\5InterviewJ_10_3_2MD: 44 - 47 (0) |  |  |
|  | This device should fit in the public, private, NGO hospitals  S002 - All interviews, discussions and observations\8InterviewJ18_6_ 2MD: 1 - 1 (0)  The paracervical block is provided in all these hospital  S002 - All interviews, discussions and observations\8InterviewJ18_6_ 2MD: 1 - 1 (0) |  |  |
| CF-C: Identify the (surgical) procedures performed | administration of paracervical block for pain control during gynecologic procedures  S002 - All interviews, discussions and observations\2InterviewS_8_1MD: 3 - 3 (0) |  |  |
|  | One of the main distinguishing features of an operating theatre is that general anaesthesia can be given and major surgeries can be done  S002 - All interviews, discussions and observations\9Interview19_7_1MD: 84 - 84 (0) |  |  |
| CF-A: Design against barriers encountered patients | Vaginas are long and so to get to a cervix (for a cervical block), you need a really long needle, like a spinal needle (not available in Kenya and expensive if you consider that you need a new one for each patient) :  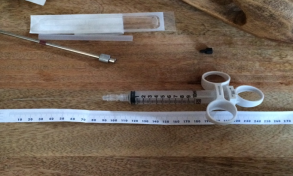  S002 - All interviews, discussions and observations\1Interview S1_7_1MD: 37 - 38 (0) | The device must be able to reach and provide pain control in the cervix/uterus. | C1 |
|  | While it is well-known that MVA causes considerable pain to a woman, it is commonly performed in Kenya (and other low-resource settings) without any analgesia due primarily to the expense of necessary equipment required to reach a woman’s cervix and administer local anesthesia  S002 - All interviews, discussions and observations\4InterviewJ_9_2_2MD: 5 - 5 (0)  In other low-income countries, local anesthesia via paracervical block is given routinely using spinal needles. However, these needles are expensive and difficult to source in low-income settings. In our experience, fear of pain during MVA deters women from seeking safe care. In addition, uncontrolled pain can cause difficulties in procedure completion as well as provider reticence with recommending and performing the procedure. Lack of access to adequate pain control  is an unacceptable limitation on a woman's right to high quality health care in pregnancy.  S002 - All interviews, discussions and observations\4InterviewJ_9_2_2MD: 5 - 6 (0) |  |  |
| CF-D: provide/organise anaesthes,sterilization | administration of paracervical block for pain control during gynecologic procedures. By attaching this device to a 10-cc syringe and standard needle, a women’s health provider will be able to inject pain medication directly into a woman’s cervix. This can relieve the pain normally felt during certain gynecologic procedures including loop electrode excision procedure (LEEP) and manual vacuum aspiration (MVA).  S002 - All interviews, discussions and observations\2InterviewS_8_1MD: 3 - 3 (0) | The device must be cleaned and sterilized using locally available methods of disinfection and sterilization. These include high-level disinfection by means of using a chemical solution or the use of pressurized steam or heat sterilization in an autoclave. | C2 |
|  | A local anaesthetic is used to numb the cervix and medicine to reduce pain and anxiety may also be offered  S002 - All interviews, discussions and observations\4InterviewJ_9_2_2MD: 2 - 2 (0)  fit standard medical procedure for cleaning for example cleaned in an autoclave.  S002 - All interviews, discussions and observations\4InterviewJ_9_2_2MD: 14 - 14 (0) |  |  |
|  | - design to used **autoclave + chemical bath** - **S002 - All interviews, discussions and observations\5InterviewJ_10_3_2MD: 20 - 20 (0)** - **Sterilize**: - Bleach and cydex? - Rural areas - High level disinfection: chemicals (2% fluoride and dip device 20min; or chloride solution for 20min and rinse with sterilised water) - Sterilization: chlorine for 10hours; or autoclave 130 degrees - S002 - All interviews, discussions and observations\5InterviewJ_10_3_2MD: 35 - 39 (0) |  |  |
|  | 1. **Cleaning + AutoClave sterilization: Metallic components, towels and gauze.** See pictures 2. Step 1: Dip in soap water for a quick wash. They use liquid soap ( i will try bring a sample to Delft) 3. Step 2: Rense with water 4. Step 3: Dip in chlorine solution for 10mins. 5. The type chlorine solution used is J**IG (Sodium hypochlorite 4-6%)** . *I will try to bring a sample to Delft* 6. *The ratio of Jig to water is 1:6.* 7. Step 4: Air dry and wrapped in cloth (wool material) 8. Step 5: Put in an autoclave (in another department 5mins away). Autoclave is set at 136 degrees celsius and 1.951Bar 9. Other items put in the autoclave are towels and gauze wraps. 10. Plastic components are almost never put in an autoclave. They are cleaning with chlorine (JIG) and CIDEX. 11. S002 - All interviews, discussions and observations\7InterviewJ13_17_5_2N_ 2MO: 8 - 17 (0) 13. **Cleaning with Water + Chlorine solution + CIDEX - Example cleaning of MVA Kits. See photo here.** 14. **Dip in Chlorine solution for 10mins. JIG (Sodium hypochlorite 4-6%).** 15. Ratio of chlorine solution to water is 200ml:6000ml see measuring cylinder 16. Cleaning in **soap water**. Hand soap that is. 17. Clean with water. 18. Immersed in CIDEX for 40mins. 19. 2.5 liters of cidex is used for over 2 weeks. 20. Clean with **distilled wate**r from the pharmacy. 21. Store in a box. 22. NB: MVA Kits have a lifespan of 2-3months. 23. One costs approx 50Euros. https://shopit.co.ke/manual-vacuum-aspiration-kit.html 24. “ We will like to use MVA equipment for more than 6months”. 25. S002 - All interviews, discussions and observations\7InterviewJ13_17_5_2N_ 2MO: 18 - 29 (0) |  |  |
|  | The device needs to be reuse and reprocessed in the healthcare facilities by the best methods available in the healthcare  facilities  S002 - All interviews, discussions and observations\8InterviewJ18_6_ 2MD: 1 - 1 (0)  Design the device to undergo chemical or  heat  sterilization  S002 - All interviews, discussions and observations\8InterviewJ18_6_ 2MD: 1 - 1 (0) |  |  |
|  | Instruments that cannot be sterilised in an autoclave are dipped in a sterilising fluid, for 20 minutes or 10 hours depending on the level of sterilisation wanted. The picture below shows an instrument that has been dipped in 2% Glutaraldehyde.  S002 - All interviews, discussions and observations\9Interview19_7_1MD: 77 - 77 (0)  once the instruments have been washed and wrapped, they are put in the autoclave machine for sterilisation. I hope this helps, I am certain that if you visit your local health facility, you will have a better perspective of these things we are talking about. 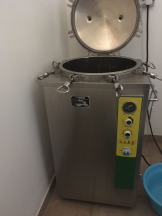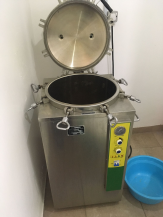  S002 - All interviews, discussions and observations\9Interview19_7_1MD: 78 - 78 (0)  After the instruments have been washed, they are wrapped and taken into an autoclave machine for sterilisation.  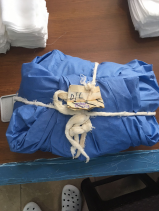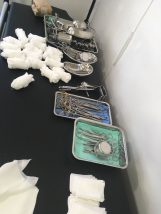  S002 - All interviews, discussions and observations\9Interview19_7_1MD: 79 - 80 (0) |  |  |
|  | terilization. This happens after the devices have been wash; soapy water, then chlorine solution and then rinsed with clean water.  We do not put plastic materials in the autoclave. Even if they can withstand the temperature  S002 - All interviews, discussions and observations\13InterviewN23_1_1MO: 1 - 1 (0) |  |  |
| CF-E: Who is part of the team providing surgery, how traine | how to extend the length of a standard syringe and needle by about 3-4 inches so that health workers can use the materials available to perform a paracervical anesthetic block  S002 - All interviews, discussions and observations\1Interview S1_7_1MD: 4 - 4 (0) | The device must be easy to use by medical personnel after having undergone training on the device use. Medical personnel include doctors, nurses and midwives, clinical offices, and anaesthetists. These medical personnel are also involved in the procurement (via the procurement department) of the device.  Design the device to be locally accessible and available. Health workers should be able to access the device and/or its related accessories locally without relying on import. | C3  C4 |
|  | procedures are widely used in low-resource countries, and are often performed by nurses or midwives in remarkably austere settings  S002 - All interviews, discussions and observations\4InterviewJ_9_2_2MD: 3 - 3 (0)  performed by mid-level providers  S002 - All interviews, discussions and observations\4InterviewJ_9_2_2MD: 5 - 5 (0) |  |  |
|  | 1. Action/procedure can be performed by multiple people (nurses, doctors, doctors with basic training/only masters) 2. S002 - All interviews, discussions and observations\5InterviewJ_10_3_2MD: 7 - 7 (0) |  |  |
| CF-F: Identify who is involved during procurement and usage | It is not intended for use by anyone other than medical personnel performing gynecologic procedures.  S002 - All interviews, discussions and observations\2InterviewS_8_1MD: 7 - 7 (0) |  |  |
|  | - This idea is not only for MVA, every procedure where the cervix needs anesthesia (such as IUB, treatment for cervix cancer, biopsy for non-pregnant internal bleeding) → but MVA highest numbers so main focus - S002 - All interviews, discussions and observations\5InterviewJ_10_3_2MD: 11 - 11 (0) |  |  |
| CF-G: Infrastructure working properly (water, electricity, etc | procedures are widely used in low-resource countries, and are often performed by nurses or midwives in remarkably austere settings, and do not rely on electricity nor require an operating theater.  S002 - All interviews, discussions and observations\4InterviewJ_9_2_2MD: 3 - 3 (0) | Ensure the device can function in areas without electrical power grid connection. | C5 |
| CF-H: Identify what other equipment is available and used. | standard syringe and needle by about 3-4 inches so that health workers can use the materials available to perform a paracervical anesthetic block  S002 - All interviews, discussions and observations\1Interview S1_7_1MD: 4 - 4 (0)  to design some kind of extender/plunger to fit to the back of a standard 10 cc syringe so that you can add the additional length you need.  S002 - All interviews, discussions and observations\1Interview S1_7_1MD: 8 - 8 (0)  The typical syringe looks like (as you probably know):  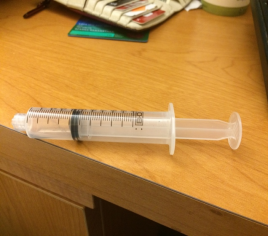  So I was wondering, why can’t you just make a plastic part like the finger holders but that would extend the whole length of the syringe to compensate for the short needle:  S002 - All interviews, discussions and observations\1Interview S1_7_1MD: 41 - 43 (0) | The device must leverage on existing medical devices such as 10-cc syringes locally available. For example, a solution that extends locally available standard 10-cc syringe with standard 18 or 22-gauge needles and provides additional length to administer paracervical block in the uterus/cervix. | C6 |
|  | attaching this device to a 10-cc syringe and standard needle, a women’s health provider will be able to inject pain medication directly into a woman’s cervix  S002 - All interviews, discussions and observations\2InterviewS_8_1MD: 3 - 3 (0) |  |  |
| IS-F: What type of accessories (consumables or reusable | standard syringe and needle  S002 - All interviews, discussions and observations\1Interview S1_7_1MD: 4 - 4 (0) |  |  |
|  | attaching this device to a 10-cc syringe and standard needle, a women’s health provider will be able to inject pain medication directly into a woman’s cervix  S002 - All interviews, discussions and observations\2InterviewS_8_1MD: 3 - 3 (0)  The pain medication used for the paracervical block (e.g. Lidocaine), the 10-cc syringe, and the standard-length needle will need to be supplied by the health facility  S002 - All interviews, discussions and observations\2InterviewS_8_1MD: 7 - 7 (0)  Other materials needed: one 10 cc syringe, one standard 18 or 22 gauge needle, 20 cc of lidocaine or alternate injectable pain medication for paracervical block  S002 - All interviews, discussions and observations\2InterviewS_8_1MD: 11 - 11 (0) |  |  |
|  | - Device must fit different type of syringes - Some different % painkiller, 10ml once and 10ml again (so device must be easy to pull out and insert again) - S002 - All interviews, discussions and observations\5InterviewJ_10_3_2MD: 15 - 16 (0) |  |  |
| IS-A: Determine if equipment will be bought, donated, or lease | Optimise to fit the market price of 5Euros  S002 - All interviews, discussions and observations\4InterviewJ_9_2_2MD: 16 - 16 (0) | Ensure the device is affordable, costing (selling price) approximately between USD4 - USD50 . This price range is comparable with the prices of other devices used in procedures requiring paracervical block. For example, a Manual Vacuum Aspiration Kit. | I1 |
|  | 1. The hospital should buy the device. We do not want to keep relying on donations. If someone donates it to us, we will take it. But we cannot rely on donations. 2. S002 - All interviews, discussions and observations\6InterviewJ_11_12_4_2MD: 30 - 30 (0) |  |  |
| IS-B: What costs are feasible? | The expected cost of the device when produced at scale would be less than 5 USD per device with an expected life span similar to that of the Karman cannula of approximately 400 procedures  S002 - All interviews, discussions and observations\3InterviewJ_9_1_2MD: 5 - 5 (0) |  |  |
|  | Optimise to fit the market price of 5Euros  S002 - All interviews, discussions and observations\4InterviewJ_9_2_2MD: 16 - 16 (0) |  |  |
|  | - 40 USD - S002 - All interviews, discussions and observations\5InterviewJ_10_3_2MD: 49 - 49 (0) |  |  |
|  | 1. The device should be approximately the same cost or lower than buying another similar device. E.g MVA kit It cost about 4000kes ($40) and does not come with a device to administer Paracervical block. 2. S002 - All interviews, discussions and observations\6InterviewJ_11_12_4_2MD: 28 - 28 (0) |  |  |
|  | costs KShs. 4000/- (Approximately 40USD)  S002 - All interviews, discussions and observations\9Interview19_7_1MD: 84 - 84 (0) |  |  |
| IS-C: What is required to make the device durable | The device may be used at room temperature and does not require any special heating or cooling precautions  S002 - All interviews, discussions and observations\2InterviewS_8_1MD: 7 - 7 (0)  The device should be stored in a clean, dry place along with the MVA kit with which it was supplied.  S002 - All interviews, discussions and observations\2InterviewS_8_1MD: 33 - 33 (0)  designed to last the lifetime of the MVA kit with which it was supplied (four hundred uses)  S002 - All interviews, discussions and observations\2InterviewS_8_1MD: 33 - 33 (0) | The device must be reusable multiple times. For example, 25 to 400 use cycles or more after disinfection and sterilization. | I2 |
|  | reusable device used to extend the length of locally available and affordable 10ml syringes  S002 - All interviews, discussions and observations\4InterviewJ_9_2_2MD: 8 - 8 (0)  Optimize to be re-used for at least 400 procedures.  S002 - All interviews, discussions and observations\4InterviewJ_9_2_2MD: 13 - 13 (0) |  |  |
|  | - cleaned and re-used; 400 times - S002 - All interviews, discussions and observations\5InterviewJ_10_3_2MD: 50 - 50 (0)  1. 25-50 procedures with sterilar (autoclave) depending on manufacturing; sterilize important against infections 2. S002 - All interviews, discussions and observations\5InterviewJ_10_3_2MD: 52 - 52 (0)  - **Use for 200 procedures** - **S002 - All interviews, discussions and observations\5InterviewJ_10_3_2MD: 54 - 54 (0)** - Usable and durable product - S002 - All interviews, discussions and observations\5InterviewJ_10_3_2MD: 70 - 70 (0) - Withstand high temperatures - S002 - All interviews, discussions and observations\5InterviewJ_10_3_2MD: 71 - 71 (0) |  |  |
|  | Durable, repaired and maintained, recycled and others  S002 - All interviews, discussions and observations\8InterviewJ18_6_ 2MD: 1 - 1 (0) |  |  |
|  | - In most settings after sterilisation the device will be stored in a sealed plastic container, here in the tropics the hottest average temperatures would be 35 degrees centigrade. - S002 - All interviews, discussions and observations\9Interview19_7_1MD: 25 - 25 (0) |  |  |
| IS-D: How will maintenance and repair be organised͍ | The device can be cleaned alongside the cannulas used in the MVA kit. Decontaminate the device for 10 minutes in 0.5% chlorine solution. Then, clean the device manually with detergent, rinse in clean water, and dry with a lint-free cloth. Next, chemically sterilize the device with 2% glutaraldehyde (CidexTM) solution by completely submerging the device in the solution for at least 10 hours. Subsequently, rinse the device thoroughly in normal saline and store in a sterile container.  S002 - All interviews, discussions and observations\2InterviewS_8_1MD: 30 - 30 (0)  The device should be stored in a clean, dry place along with the MVA kit with which it was supplied. There is no maintenance required for this device. It is designed to last the lifetime of the MVA kit with which it was supplied (four hundred uses).  S002 - All interviews, discussions and observations\2InterviewS_8_1MD: 33 - 33 (0) | Design the device such that after-sales services can be provided. This includes providing spare parts for repair, maintenance, and upgrade. Or in other cases, recovery (that is through recontextualization, refurbishment and remanufacturing) and/or recycling of obsolete parts.  Ensure the device must be manufactured through locally available large-scale or decentralised manufacturing processes that support local after-sale services.  Ensure the device can be included and sold together with other existing devices used in procedures requiring paracervical block. For example, the device could be sold in a pack with 10-cc syringes or sold in a pack with existing MVA kits. | I3  I4  I5 |
| IS-E: Relation btw providers and hospital usage and disposal | 1. **Maybe extend the syringe and not the needle since we cannot locally manufacture precision needles in Kenya.** 2. **S002 - All interviews, discussions and observations\1Interview S1_7_1MD: 17 - 17 (0)** 3. we are going to formally test it with about 20 providers a 4. S002 - All interviews, discussions and observations\1Interview S1_7_1MD: 276 - 276 (0) 5. The Local Blacksmith - Digital Fabrication for user products for development 6. S002 - All interviews, discussions and observations\1Interview S1_7_1MD: 293 - 293 (0) |  |  |
|  | design and manufacturing scheme for replicability in a low-resource setting such as Kenya, while also guaranteeing optimal quality and leveraging existing manufacturing capabilities available locally.  S002 - All interviews, discussions and observations\3InterviewJ_9_1_2MD: 19 - 19 (0) |  |  |
|  | - Contacted NGO, for now they did it themselves because it takes longer through a NGO, but probably have them on board later - S002 - All interviews, discussions and observations\5InterviewJ_10_3_2MD: 64 - 64 (0) |  |  |
|  | 1. The hospital already buys these MVA kits. You can partner with an MVA kit supplier so the paracervical block device is bought with the MVA KIT. 2. S002 - All interviews, discussions and observations\6InterviewJ_11_12_4_2MD: 29 - 29 (0) |  |  |
|  | - eventually convince the manufacturers of the MVA kit to include the Chloe SED as part of the Kit. The most popular kit in the Kenyan market is the IPAS Kit (DKT manufactured), the manufacturer recommends maximum use of 25 times, but typically it is used many more times over. There are anecdotal reports of 400 uses. - S002 - All interviews, discussions and observations\9Interview19_7_1MD: 42 - 42 (0) |  |  |
|  | We always need spare parts. We havest spare parts from other similar devices. Suppliers should ensure spare parts can be supplied  S002 - All interviews, discussions and observations\14InterviewB24_1_24MO: 1 - 1 (0)  Device providers should ensure broken devices are recovered.  Also suppliers of device should prvide support for repair, provisionof spareparts, maintenance or upgrade. These device will break at some point.  S002 - All interviews, discussions and observations\14InterviewB24_1_24MO: 1 - 1 (0)  Broken devices are disposed in the dust been. Maybe companies can recolllect them and recycle  S002 - All interviews, discussions and observations\14InterviewB24_1_24MO: 1 - 1 (0) |  |  |

# Supplementary Data 2 - Establishment of forces required for use on Syringe Extension Device

**Case: Syringe Extension Device.**

A syringe extension device (See Figure 1) has been designed to support the administration of paracervical analgesia during ganocological procedures in Kenya. It consists of three homogenous plastic parts, which are easily assembled and disassembled. That is, it is done without the use of any fasteners, glue, snap fits etc. The syringe extension device will be used to draw-in and inject a total of 20mL of 1% lidocaine (1) using a 10ml syringe. 2ml of  1% lidocaine is  first injected into the superficially into the anterior lip of the cervix and the  remaining lidocaine in equal amounts  (approximtae 4.5ml) at the cervicovaginal junction, at 2, 4, 8 and 10 o’clock Grasp cervix with the tenaculum (1). Existing research that show the force required to draw-in  or  inject (i.e push-out) lidocaine into a syringe is missing in literature. Knowledge of how much force is needed to draw-in  or  inject (i.e push-out) lidocaine into a syringe is critical to establishing the maximum force to be exerted on the syringe extension device device without causing the device to fail, excessively deform or otherwise be rendered ineffective.


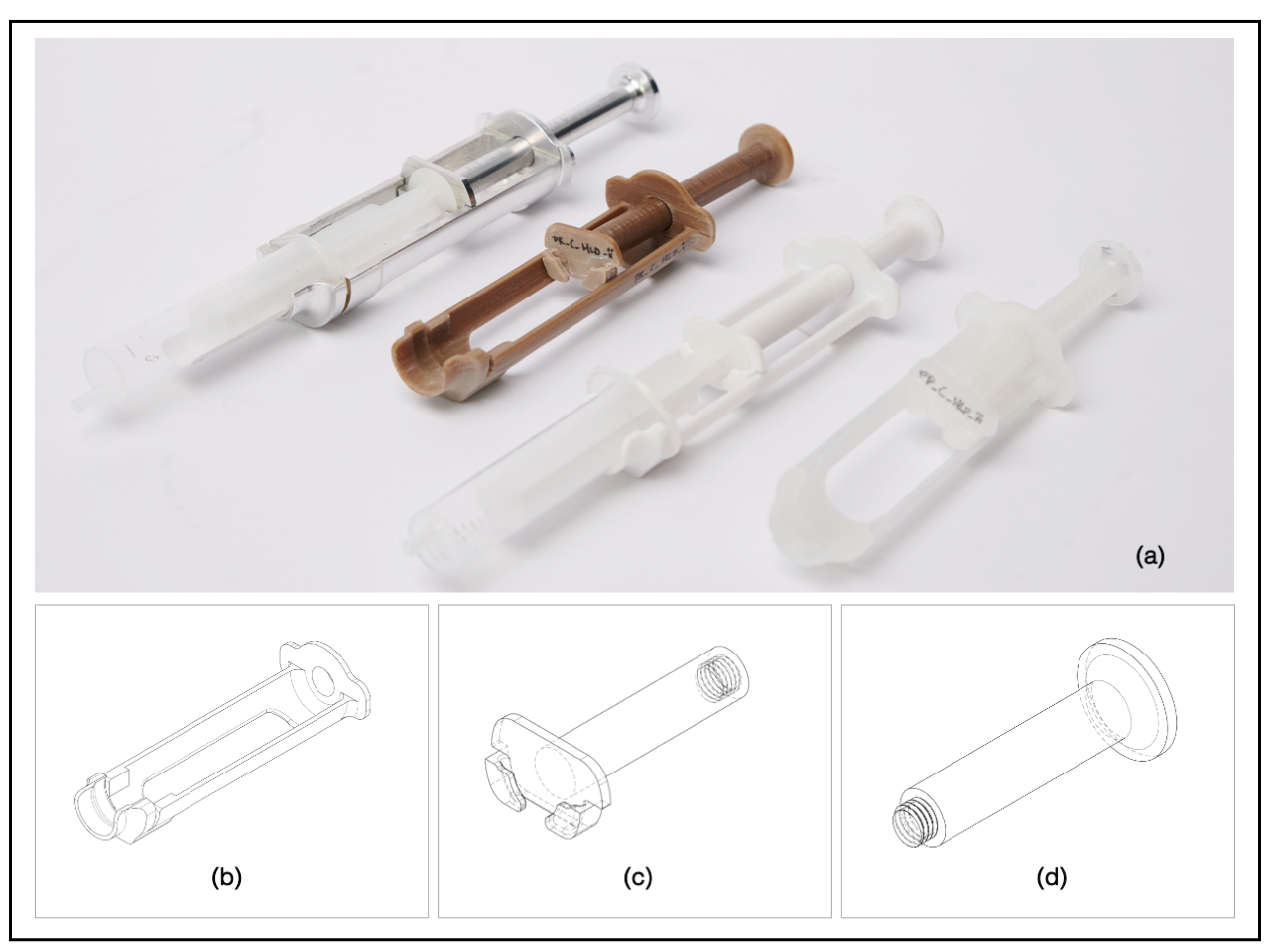
 **Supplementary Data 2 - Figure 1:** Syringe extension device for provision of paracervical block. (a) from Left to right is a the syringe extension device in manufactured in alluminium, PEEK, PP and attached with a attached with a 10cc syringe and lastly is PP syringe without a 10cc syringe. (b), (c) and (d) are the individual parts of (a). Where (b) is the body, (c) is the plunger and (d) is the thumb

**Experiment Aim**

Establish the amount of force (in N, where N is Newtons)  required to  draw-in  or  inject (i.e push-out) liquid pain medication from a 10cc syringe while using the a Syringe Extension Device for paracervical block.

**Experiment Setup**

For testing the force on Syringe Extension Device the setup  in **Supplementary Data 2 - Figure 2** (**Supplementary Data 2 – Table 1** shows the technical specification apparatus used) was established. The setup in Figure 2 pushes out (inject) water from a 10cc syringe attached to Syringe Extension Device at constant speed. In another case, setup in Figure 2 was used to draw-in water from a 10cc syringe attached to Syringe Extension Device. Water was used as a substitute due to lack of availability of 1% lidocaine solution. Note that 1% Lidocaine is mixed in water though other substances are added to it to make it isotonic and to make the PH  right for the body (2). As such we assumed that the flow of water through the syringe will be of the same characteristic if 1% Lidocaine was used.


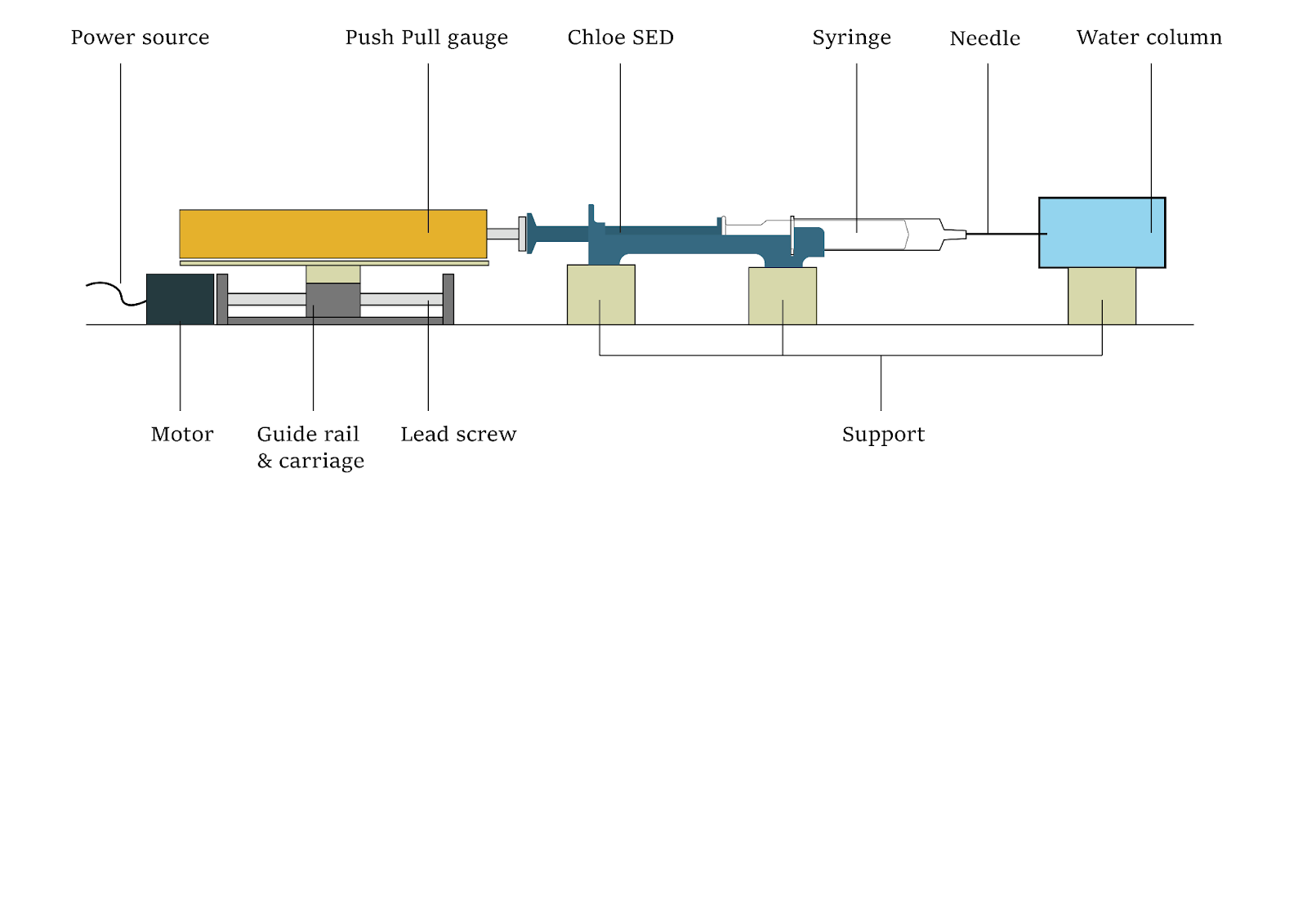


**Supplementary Data 2 - Figure 2:** Schematic of setup

**Supplementary Data 2 – Table 1**: Technical specifications of the apparatus use during the setup as seen in Supplementary Data 2 - Figure 2.

| **Apparatus** | **Make / model** |
| --- | --- |
| Force gauge | Success, Japan/ MODEL ANF- 200 |
| Motor | NEMA 11 - 20x20mm - 28h30h0604a2 |
| Microcontroller | Seeeduino Lotus |
| Driver | DRV8825 |
| Syringe | BD Plastipak^TM^, 10mL |
| Needle | BD Microlance^TM^ 3, 21G 2”, REF 301155 |

The water in the syringe was pushed out (inject) or drawn-into the syringe at constant speed established based on flow rates. Two different flow rates were used for pushes out (inject) or drawn-into water out of the syringe . That is, a slow flow rate of 5 ml/min and a fast flow rate of and 15 ml/min as recommended by (3,4). The motors used in the experiment set up (see **Supplementary Data 2 - Figure 2**) were adjusted electronically (see **Supplementary Data 2 - Figure 3**) to produce the respective flow rates of 5 ml/min and 15 ml/min used to measure the force (with a force guage) when pushes out (inject) or drawn-in liquid into the syringe that is attached to the Syringe Extension Device.


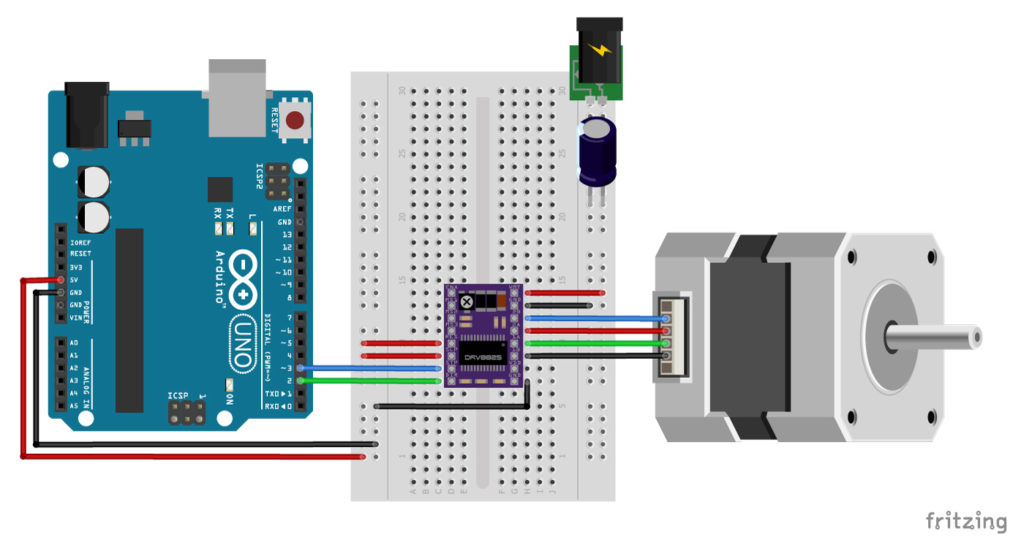


**Supplementary Data 2 – Figure 3**. Electronics Schematics use to control the flow rate (5).

The established flow rate is used to push-out (inject) water into water column of 109 mm as seen in Figure 4. The water column of 109 mm was use to mimic injecting pain medication into the cervix. Also the water colum of 109mm (whic is equal to 8mmHg)  was use to produce the pressure in the cervix. Note that pressure in the cervix estimated be comparable with normal pressure of a tissue compartment which falls between 0 and 8 mmHg(6). Similar sincel lidocaine is highly regularised drug and difficult to purcshe of the shelf for this experiment, a water column of 20ml of water was used instead. Besides, the density of lidocaine and water are the same (7,8) and thus waas suitable to mimic the case of drawing-in  1% of 20ml Lidocaine into the syringe. Note the entire setup as seen in Figure 2 and 4 was placed horizontalliy for guarantee stability.


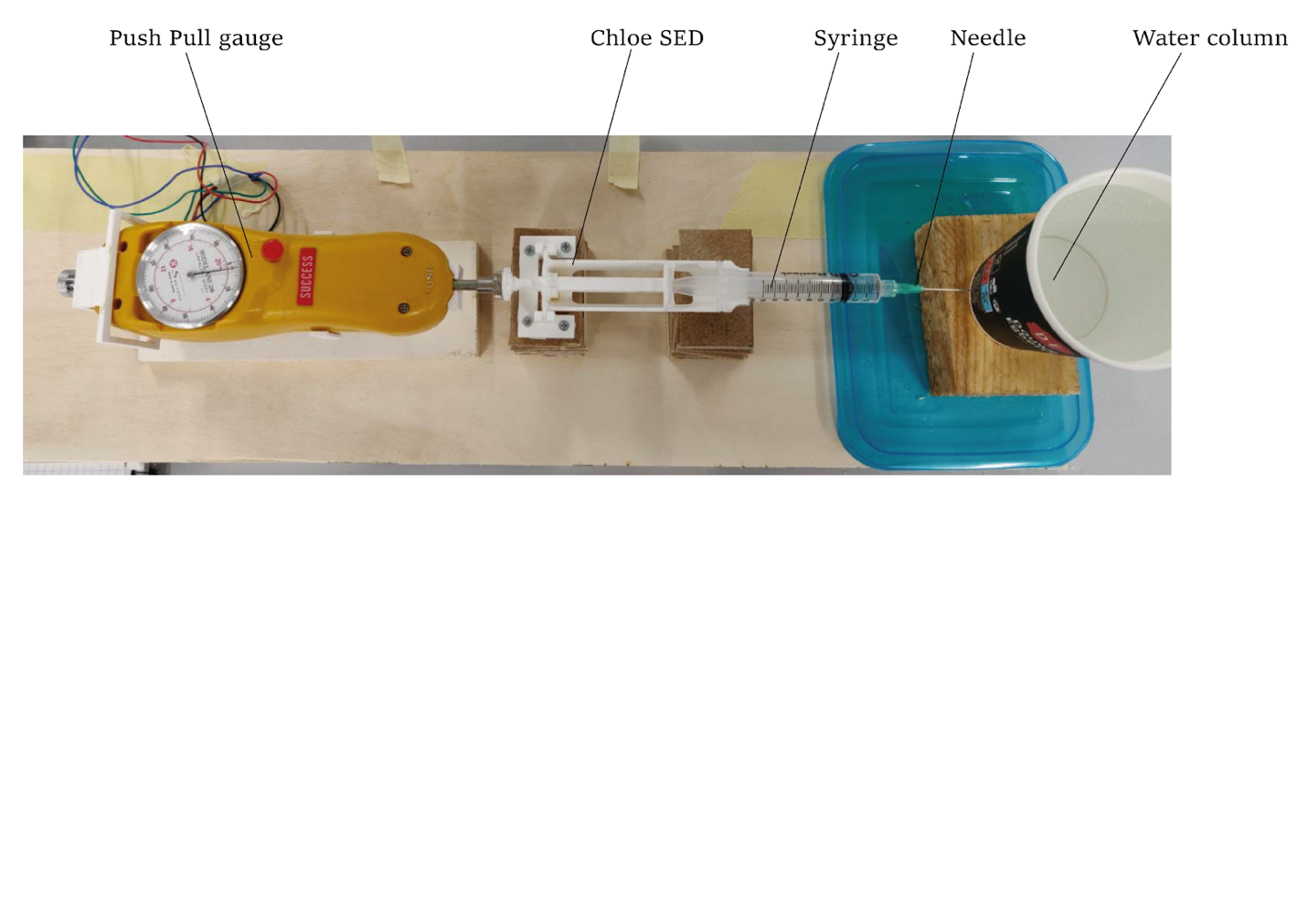
 **Supplementary Data 2 - Figure 4**. Setup of the experiment in practice.

**Step by Step Procedure.**

**Step 1:** For measuring the pushes out (inject) force the following step was performed.

1. Draw in 2ml of water into the syringe. This represents the 2ml of  the 20ml 1% lidocaine is  first injected into the superficially into the anterior lip of the cervix (1).
2. Assemble electronics (i.e arduino, motor and driver) as per Figure 3.
3. Assemble the electronics, the Syringe Extension Device, syringe (filled in with 2ml of water)  and needle setup according to **Supplementary Data 2 - Figure 4**.
4. Next, ensure the force gage is reset to zero.
5. Power on the electronics.
6. Set the electronics to produce speed required to drive the system at flow rate of  5ml/min and a fast flow rate of and 15 ml/min and run. Ensure the syringe is injected into the water colum 109 mm.
7. Record the force value.
8. Disconnect power source and reset the force gauge.
9. Repeat the steps 9 more times so as to established different set of values for averaging.
10. Repeat **Step 1** (from 1-9) for 5ml. This represents the  remaining lidocaine provided in equal amounts  (approximtae 4.5ml) at the cervicovaginal junction, at 2, 4, 8 and 10 o’clock when grabbing the cervix with the tenaculum (1).

**Step 2:** For measuring the draw-in  (draing in liquid into the syringe) force, the following step was performed.

1. Assemble electronics (i.e arduino, motor and driver) as per **Supplementary Data 2 – Figure 3**.
2. Assemble the electronics, the Syringe Extension Device, syringe (empty syringe)  and needle setup according to Figure 4.
3. Next, ensure the force gage is reset to zero.
4. Power on the electronics.
5. Set the electronics to produce speed required to drive the system at flow rate of  5ml/min and a fast flow rate of and 15 ml/min and run. Ensure the syringe is inserted into 20ml container as seen in
6. Draw in 2ml of water in the syringe
7. Record the force value.
8. Disconnect power source and reset the force gauge..
9. Repeat the steps 9 more times so as to established different set of values for averaging.
10. Repeat **Step 2** (from 6-9) while drawing 5ml of liquid into the syringe

**Results.**

A total of 80 cycles of the test were completed**.** A minimum value of of 4N and maximum value of 8N was recorder, when injecting or drawing in 2ml and 5ml liquid into the syring at either fast or slow rates as seen n **Supplementary Data 2 - Table 2.**

**Supplementary Data 2 - Table 2: Forces established in inject or push out water through a syringe attached to the Syringe Extension Device.**

|  | Inject (push out water from the Syringe) | | | | Draw-in (pull or draw in water  into the syringe) | | | |
| --- | --- | --- | --- | --- | --- | --- | --- | --- |
|  | Fast | Slow | Fast | Slow | Fast | Slow | Fast | Slow |
|  | 2mL | | 5 mL | | 2mL | | 5 mL | |
| [S.No](http://s.no/) | Case 1 | Case 2 | Case 3 | Case 4 | Case 5 | Case 6 | Case 7 | Case 8 |
| 1 | 8 | 6 | 7 | 6 | 8 | 6 | 8 | 6 |
| 2 | 8 | 5 | 7 | 7 | 8 | 6 | 8 | 6 |
| 3 | 7 | 6 | 7 | 7 | 8 | 6 | 8 | 6 |
| 4 | 7 | 6 | 7 | 8 | 8 | 6 | 8 | 6 |
| 5 | 7 | 5 | 7 | 8 | 8 | 6 | 8 | 6 |
| 6 | 6 | 5 | 7 | 8 | 8 | 6 | 8 | 6 |
| 7 | 7 | 4 | 7 | 8 | 8 | 6 | 8 | 6 |
| 8 | 7 | 6 | 7 | 8 | 8 | 6 | 8 | 7 |
| 9 | 6 | 6 | 8 | 8 | 8 | 6 | 8 | 6 |
| 10 | 7 | 6 | 7 | 8 | 8 | 6 | 8 | 6 |
| Average | 7 | 5.5 | 7.1 | 7.6 | 8 | 6 | 8 | 6.1 |

**Discussion**

The overall maximum injection force of  8N (average Max is 7.6N)  and the maximum force during withdrawing of 8 N (average Max is 8N) was recorded. The forces are measured keeping the entire system horizontal. In reality the syringe is not always perfectly horizontal, there is variation during injection and the needle is usually pointing a little upwards during withdrawal. This means that less force might be required to draw-ing liquid into the syringe, though a safety factor to ensure the device works well beyond what it can handle is also recommended. As such taking into a safety factor of 3 as recommended by for medical devices, (9), the maximum injection (push out liquid) or draw-in force of 24N (calcaluated as 8N x factor of safety of 3) should be considered.

**Conclusion**

Bases on the above experiment, it can be concluded that a maximum force between **24N** can be exerted on the syringe to pull in (draw the medication into the syringe) or inject (push out) pain medication such as lidocaine during paracervical block. This force should be able to be used on the device without causing the device to deform, fail or otherwise be rendered ineffective.

# Supplementary Data 3 - Life cycle assessment calculation script performed using Activity Browser software, which builds on brightway2 python package for LCA calculations.

1. Device Weight

The weight of Chloe SED^®^ in PP, PEEK, and aluminium is 151 grams, 220 grams and 451 grams respectively.

1. Materials and manufacturing.

Because the exact intended manufacturing materials (Homopolymer PP, PEEK and aluminium-6061 grade) or production process (injection moulding) was not available in the Ecoinvent v3.9.1 database, proxy materials and processes were used. Granulate Polypropylene (PP) and aluminium wrought alloy were used as alternatives to Homopolymer PP, and aluminium-6061 grade. PP pipe extrusion and section bar extrusion was used as alternation production techniques for injection moulding the final design in PP and aluminium respectively. End-of-Life is not taken into account due to the complexity of defining the exact End-of-Life pathways in the local context.

1. We anticipate 500 Paracervical Block clinical procedures per year.
2. Yearly material consumption of a clinic:
   1. We assume roughly 500 clinical operations in one yea
   2. Based on the clinical operation in (3) and reuse cycle in **Supplementary Data 3 – Table 1** below, the consumption of 0.5 units of aluminium Chloe SED^®^ and 20 in PEEK per year is needed when using either autoclave or chemical cleaning methods.
   3. The consumption of 100 devices or 20 Chloe SED^®^ devices in PP is needed per year when using either autoclave or chemical cleaning methods, respectively.

| **Supplementary Data 3 – Table 1**: Number of devices needed as per the LCA functional unit. | | | | |
| --- | --- | --- | --- | --- |
| **Chloe SED^®^ Device type (in material)** | **Device weight (grams)** | **Reprocessing method and reuse cycles** | | **Number of devices needed per year as per the functional unit** |
|  |  | **Method** | **Estimated ruse cycles as per material type** |  |
| Homopolymer Polypropylene (PP) | 151 | Autoclaving | 5 (10,11) | 100 |
|  |  | Chemical sterilisation | 25 (12–14) | 20 |
| Polyetheretherketone (PEEK) | 220 | Autoclaving | 25 (15) | 20 |
|  |  | Chemical sterilisation |  |  |
| Aluminium (6061 grade) | 451 | Autoclaving | 1000 (16) | 0.5 |
|  |  | Chemical sterilisation |  |  |

1. Reprocessing using chemical sterilisation.

This is calculated for cleaning a batch of 8-10 devices in a hospital in LRS in Kenya. We choose 10 devices, and it is the same for PP, PEEK, and aluminium. All inputs per cleaning session are multiplied by 50 (500 procedures per year/batch of 10 Chloe SED^®^). Cleaning Chloe SED^®^  by chemical sterilisation requires the following raw materials.

- 1. 0.5% chlorine solution is needed and dispose of after use. This solution comprises of 10 liters, so 50 ml of chlorine = 78 grams of chlorine (density of 1.56 kg chlorine per liter chlorine) = 78 * 50 = 3.9 kg.
  2. Rinse with cool water (10 litres) and dispose after use (every cycle) = 500 kg.
  3. Wash with detergent water. That is Omo hand washing powder 30g in 10 litres of water = 1.5kg. Dispose of after-use (modelled as generic cleaning consumable, disposal not modelled).
  4. Dip in Sodium hypochlorite (alternative to 2% glutaraldehyde).
     1. 1 cycle a day, so we can do 14 cycles with this amount of material). That is 50/14 = 3.57, this is the factor we multiply with to get to 500 cycles per year.
  5. Dip in 10 liters sodium hypochlorite for 10hrs (200 ml of 6% sodium hypochlorite (1.11 g/cm³ density) in 600 ml of water => 12.5 * 200ml = 2.5L * 0.06 = 150ml * 1.11 * 3.57 = 594.4 grams pure sodium hypochlorite consumed per year). Dispose of the solution after 14 days (disposal not modelled).
  6. Rinse in sterile water (10 liters) – Dispose of it after 14 days. (once per 14 days = 35.7 kg water).

1. Reprocessing using Autoclave sterilisation.

In reality, autoclave sterilisation is done for a batch of about 20-25 devices. We choose 25 devices, so 20 cleaning cycles per year of operation. Note that PP syringes last only 5 usages with autoclave cleaning compared to 25 in chemical cleaning. The autoclave procedure includes the following raw materials. Note that all inputs per cleaning session are multiplied by 20 (500 procedures per year/batch of 25 Chloe SED^®^).

- 1. 0.5% chlorine solution. Dispose of after-use (disposal not modelled). 10 liters, so 50 ml of chlorine = 78 grams of chlorine (1.56 kg per litre) = 78 * 20 = 1.56kg.
  2. Rinse with cool water (10 litres). Dispose of after use. 200 kg.
  3. Put in Autoclave - 136 degrees celsius and 1.951Bar (Sliding single door horizontal) for 1.5-2hrs. 7 kwh per cycle, for 40 kg of material.
     1. For PP the calculation is as follows, 1 syringe => 151/40.000 grams = 0.003775 * 7 kwh * 500 uses =13.21 kwh.
     2. For Aluminium the calculation is as follows, 1 syringe => 451 / 40.000 grams * 7 * 500 = 39.46 kwh.
     3. For PEEK the calculation is as follows, 1 syringe => 220/40.000 grams = 0.0055 * 7 kwh * 500 uses =19.25 kwh.

**Supplementary Data 3 – Table 1**:Data points sourced from ecoinvent 3.9.1

| **Reference product** | | **amount** | **unit** | **IPCC 2021 \| climate change \| global warming potential (GWP100)** | |
| --- | --- | --- | --- | --- | --- |
| **0** | One year clinic (aluminium, autoclave) | 1 | Year | 15.50 | Kg CO2 |
| **1** | One year clinic (aluminium, chemical) | 1 | Year | 17.46 | Kg CO2 |
| **2** | One year clinic (PEEK, autoclave) | 1 | Year | 86.20 | Kg CO2 |
| **3** | One year clinic (PEEK, chemical) | 1 | Year | 92.93 | Kg CO2 |
| **4** | One year clinic (PP, autoclave) | 1 | Year | 48.24 | Kg CO2 |
| **5** | One year clinic (PP, chemical) | 1 | Year | 22.62 | Kg CO2 |
|  |  |  |  |  |  |
| **Material** | | | | | |
| **6** | aluminium, wrought alloy | 1 | kilogram | 13.83 | Kg CO2 |
| **7** | PEEK | 1 | kilogram | 17.40 | Kg CO2 |
| **8** | polypropylene, granulate | 1 | kilogram | 2.30 | Kg CO2 |
|  |  |  |  |  |  |
| **Impact fot 1 Chloe SED** | | | | | |
| **9** | Syringe (aluminium) | 1 | unit | 6.59 | Kg CO2 |
| **10** | Syringe (PEEK) | 1 | unit | 3.94 | Kg CO2 |
| **11** | Syringe (PP) \| | 1 | unit | 0.42 | Kg CO2 |
|  |  |  |  |  |  |
| **Only the sterilisation (for one entire year)** | |  |  |  |  |
| **12** | Autoclave sterilisation (Aluminium) | 1 | year | 12.21 | Kg CO2 |
| **13** | Autoclave sterilisation (PEEK) | 1 | year | 7.43 | Kg CO2 |
| **14** | Autoclave sterilisation (PP) | 1 | year | 6.01 | Kg CO2 |
| **15** | Chemical sterilisation | 1 | year | 14.17 | Kg CO2 |

# Supplementary Data 4 – 10cc syringes snapped onto Chloe SED^®^

Fifteen 10-cc syringes were collected from healthcare facilities in the local context (Kenya) and used on the Syringe Extension Device.


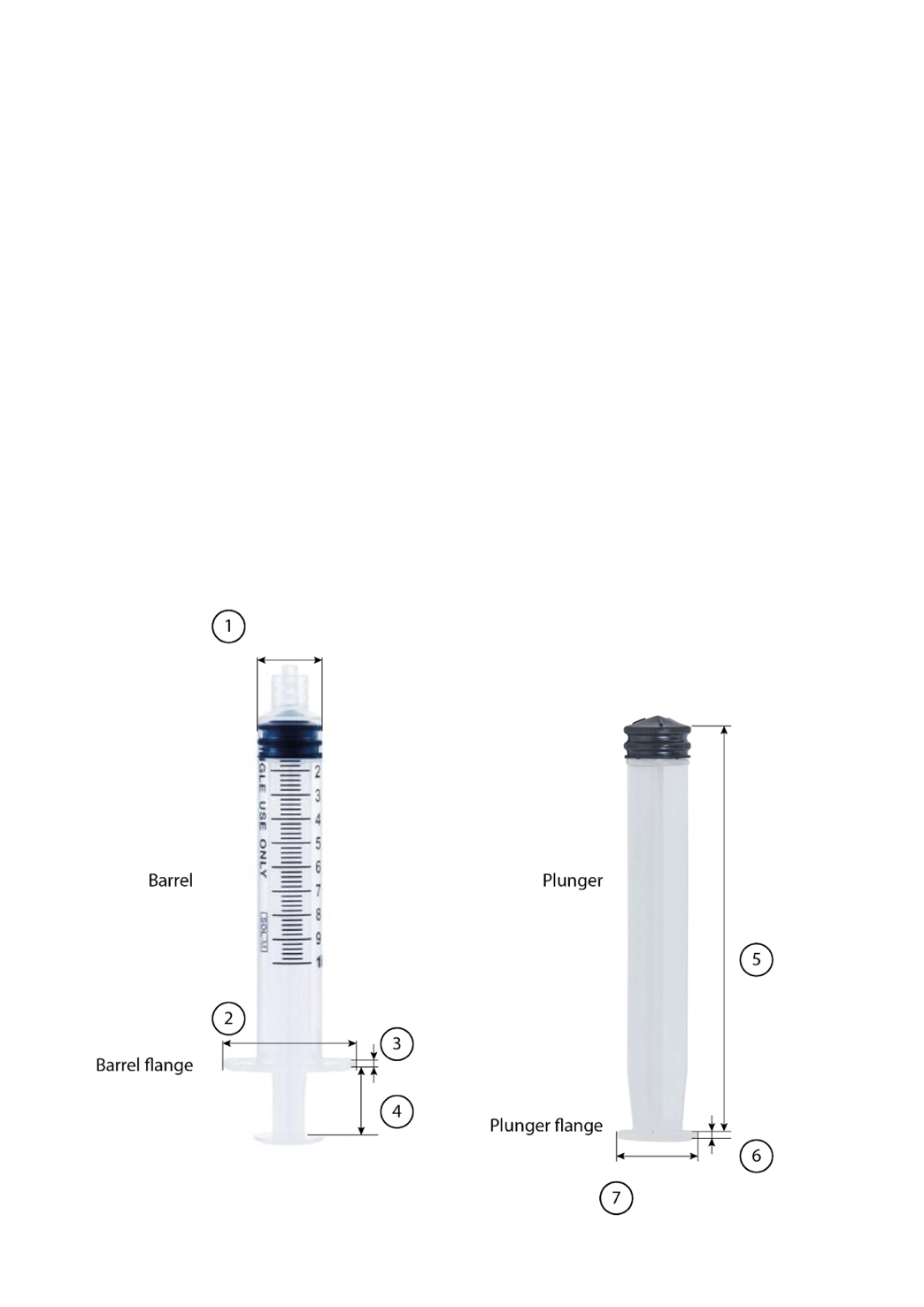

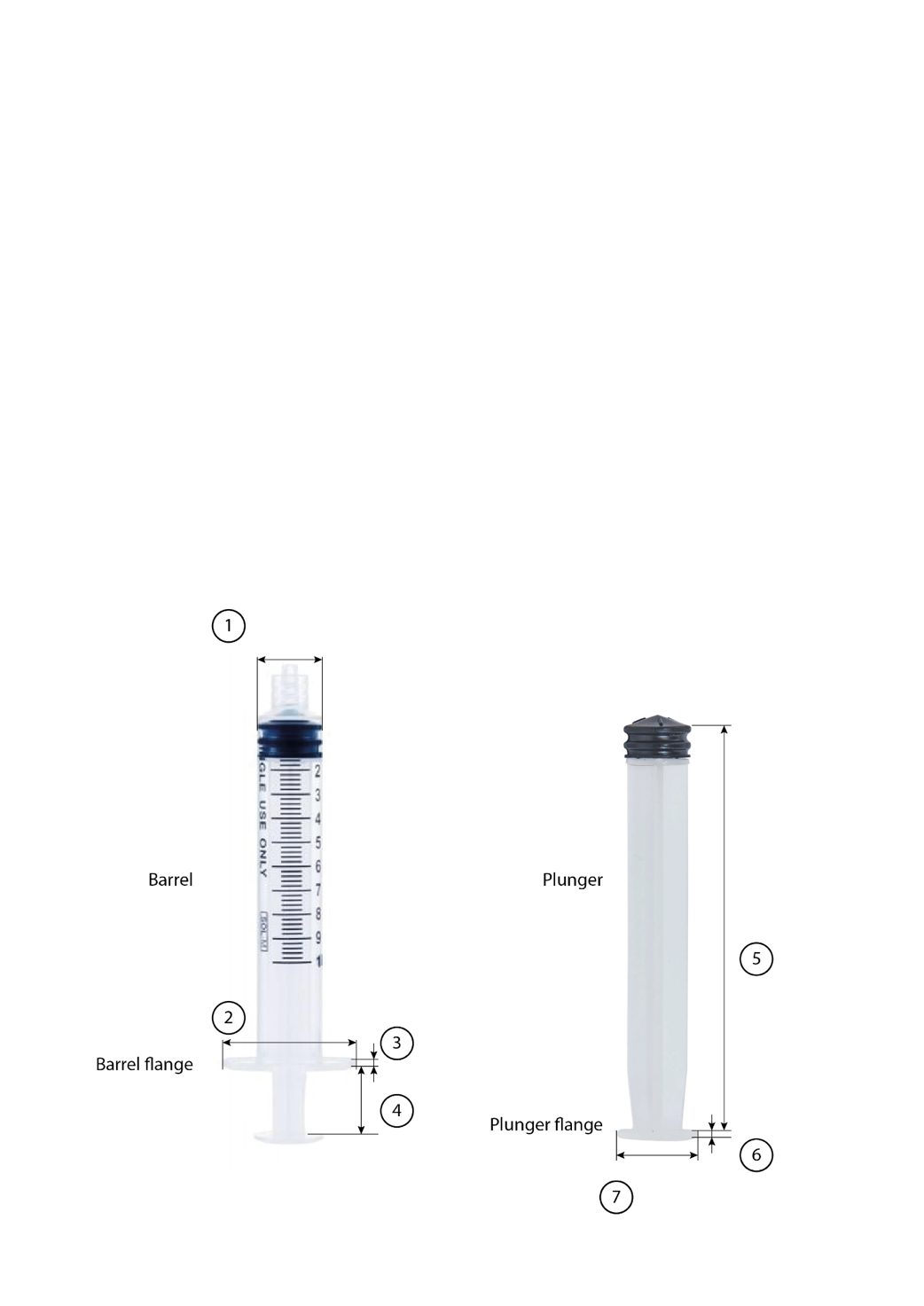


1. Barrel diameter
2. Barrel flange width
3. Barrel flange thickness
4. Stop distance
5. Plunger length
6. Plunger flange thickness
7. Plunger flange diameter

Supplementary Data 4 – Table 1. Fourteen syringe samples sourced from the local context in Kenya.

| 1 | \| 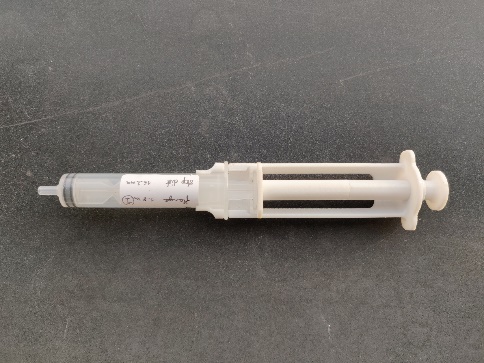 \| \| --- \| | 2 | \| 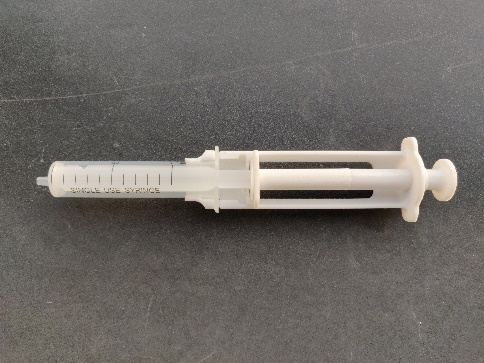 \| \| --- \| |
| --- | --- | --- | --- | --- | --- |
| 3 | 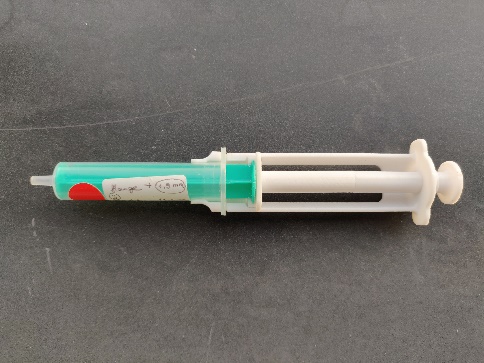 | 4 | 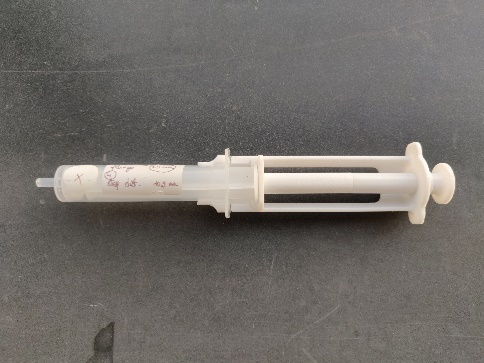 |
| 5 | 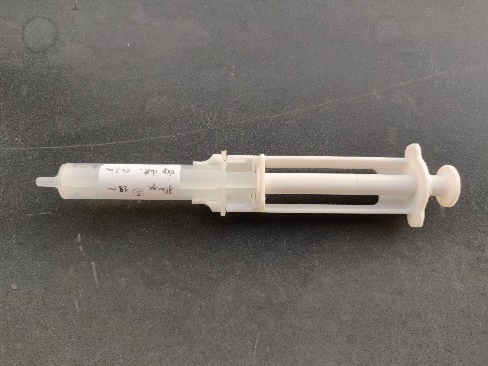 | 6 | \| 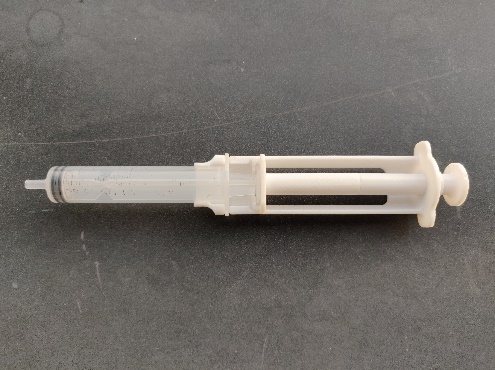 \| \| --- \| |
| 7 | 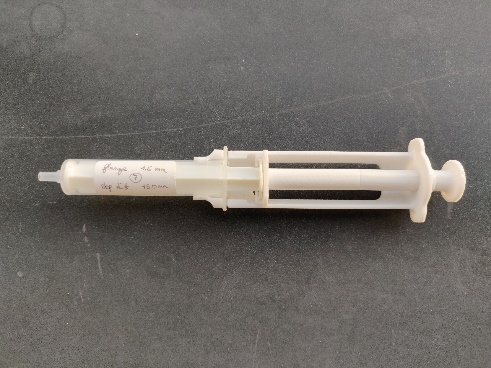 | 8 | 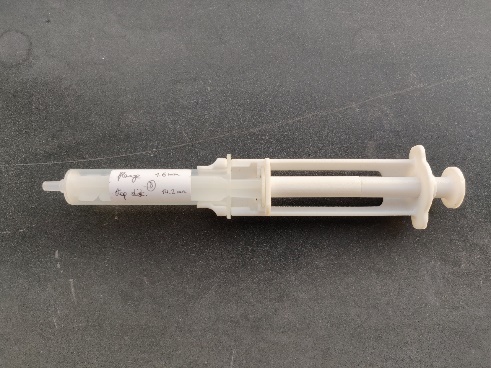 |
| 9 | 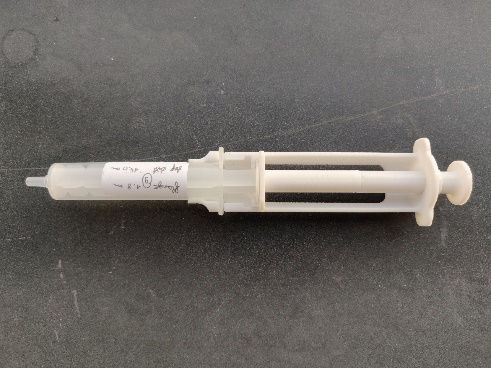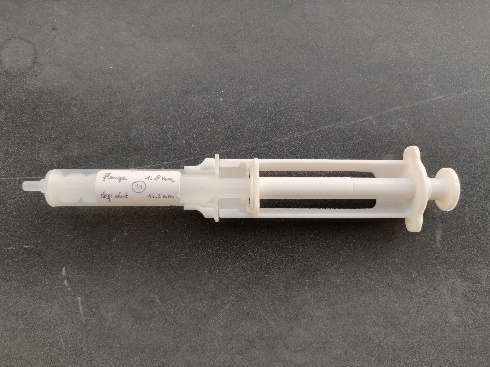 | 10 | 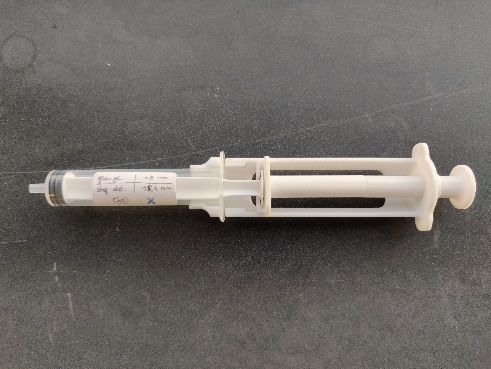 |
| 11 |  | 12 | 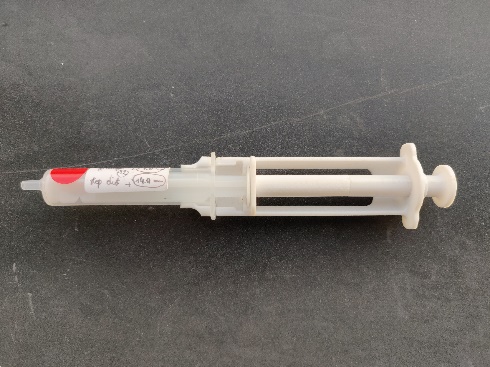 |
| 13 | 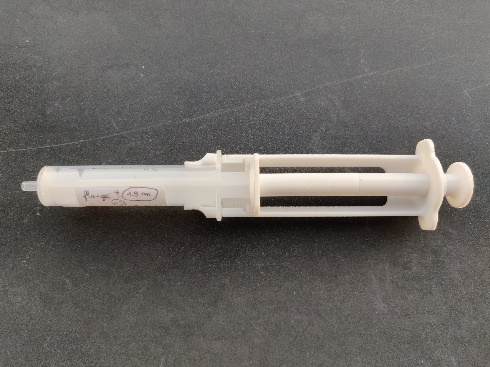 | 14 | 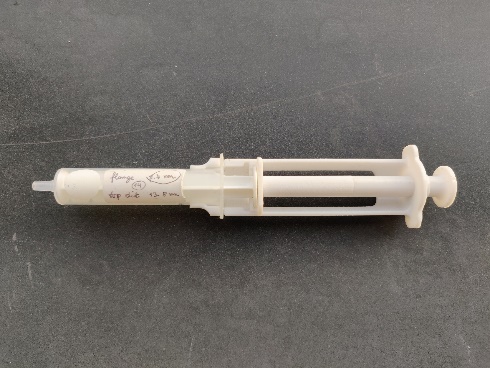 |
| 15 | 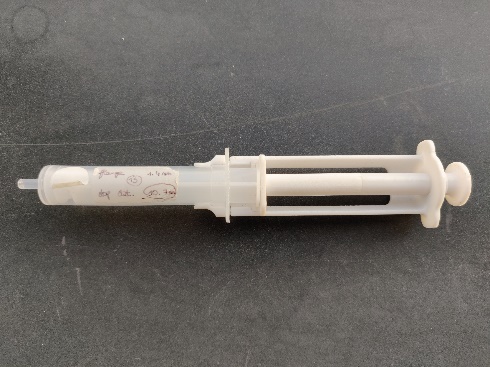   \|  \| \| --- \| |  |  |

**Supplementary Data 4 – Table 2**: Measurement of syringes in Supplementary Data 4 – Table 1

|  | Barrel | | | | Plunger | | |
| --- | --- | --- | --- | --- | --- | --- | --- |
| S.No. | Barrel Diameter | Barrel flange width | Barrel flange thickness | Stop distance | Plunger Flange diameter | Plunger Flange thickness | Plunger length |
| 1 | 16.4 | 28.8 | 1.8 | 16.2 | 17.7 | 2 | 90.92 |
| 2 | 17 | 31 | 1.6 | 13.4 | 19.8 | 1.5 | 90 |
| 3 | 17.4 | 30.8 | 1.9 | 11.6 | 19.8 | 1.7 | 88.54 |
| 4 | 17.4-18.4 | 32.7 | 1.3 | 10.9 | 19 | 1.6 | 90.4 |
| 5 | 16.8 | 30 | 1.8 | 14.2 | 19.4 | 1.6 | 89.88 |
| 6 | 16.9 | 30 | 1.8 | 14 | 19.2 | 1.6 | 88 |
| 7 | 16.8 | 29.9 | 1.6 | 15 | 19.9 | 1.9 | 90 |
| 8 | 16.8 | 30 | 1.6 | 14.2 | 19.4 | 1.6 | 90.34 |
| 9 | 16.8 | 30 | 1.8 | 14 | 19.4 | 1.6 | 89.96 |
| 10 | 16.4 | 31.4 | 1.5 | 16.6 | 15.9 | 1.4 | 94 |
| 11 | 16.9 | 30.3 | 1.8 | 14.2 | 19.5 | 1.9 | 90 |
| 12 | 17 | 30 | 1.9 | 14 | 19.5 | 1.6 | 89.98 |
| 13 | 17.4 | 30 | 1.9 | 12.2 | 20.2 | 2 | 88 |
| 14 | 16.8 | 29.9 | 1.4 | 13.8 | 19.8 | 1.9 | 89.94 |
| 15 | 17.5-18.4 | 32.4 | 1.4 | 10.7 | 19.3 | 1.6 | 90.4 |

# Supplementary Data 5 - Von Misses stresses and the corresponding displacement for PEEK and Aluminium.


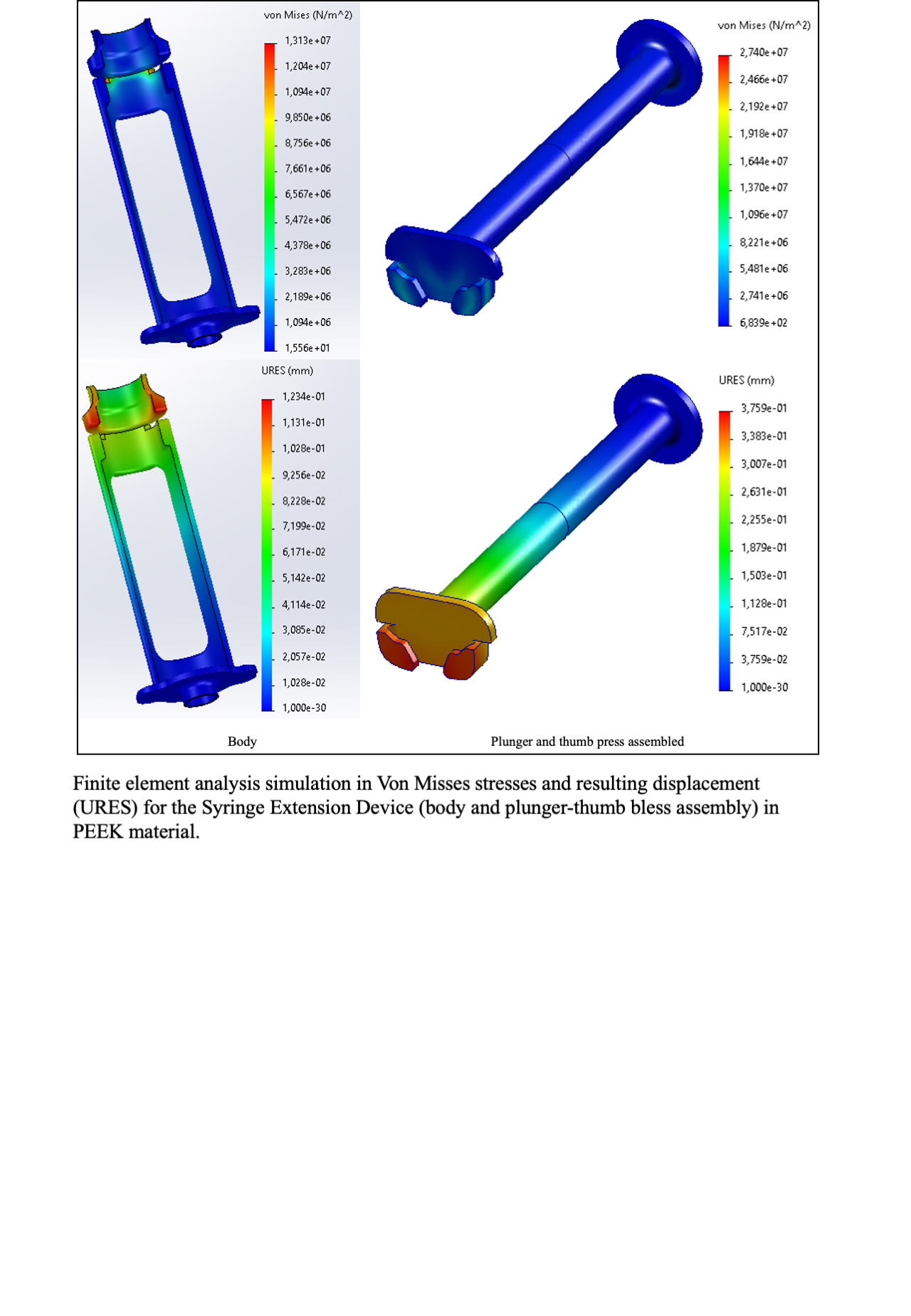


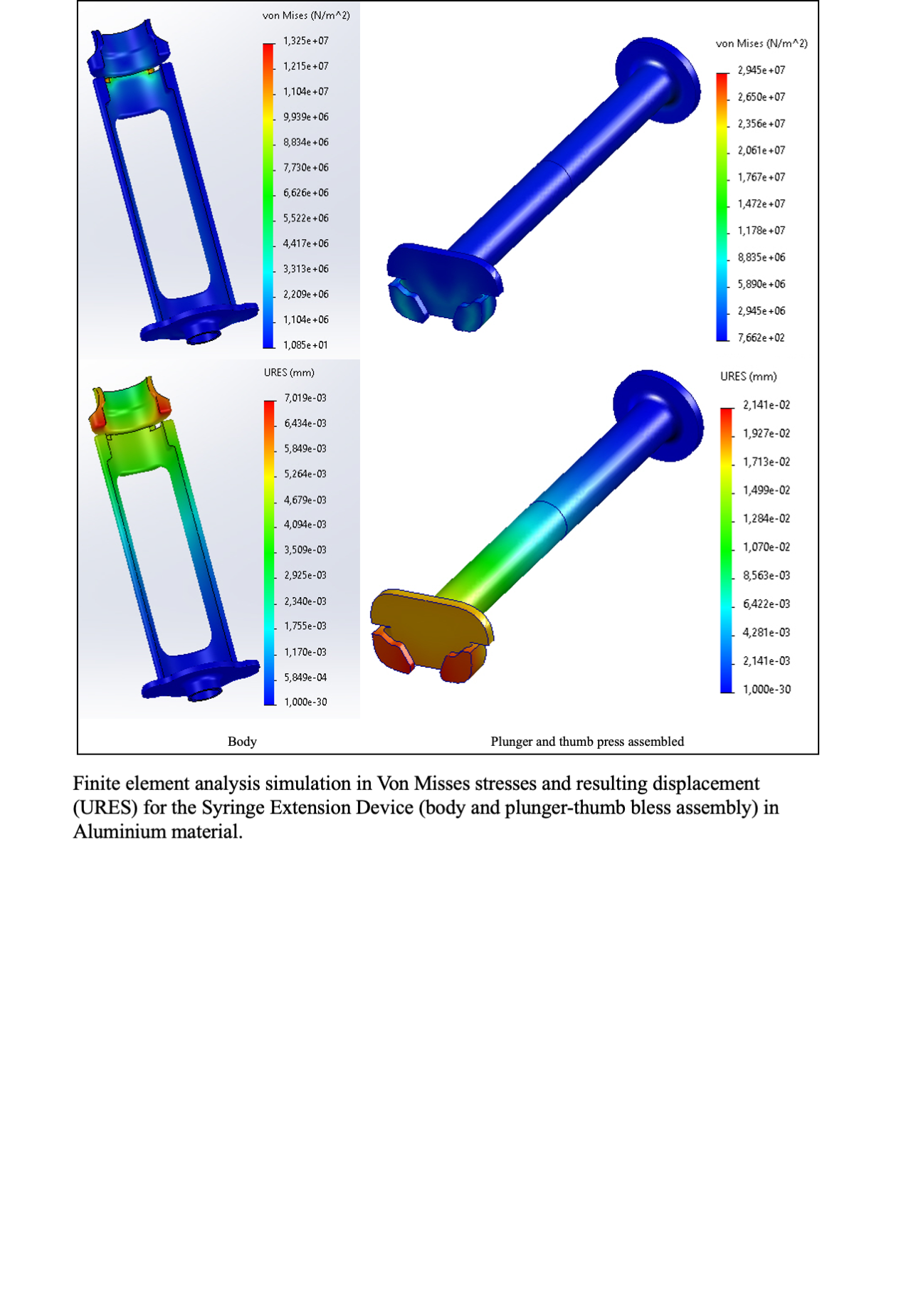


**References**

1. IPAS. Paracervical Block Technique [Internet]. 2021 [cited 2023 Jan 5]. Available from: https://www.ipas.org/wp-content/uploads/2021/06/Paracervical-Block-Technique-PARABLK-E21.pdf

2. Time of Care. Drug Dilutions, Clearly Explained | Time of Care [Internet]. 2017 [cited 2023 Jan 5]. Available from: https://www.timeofcare.com/drug-dilutions-clearly-explained/

3. Patil JJ, Ford S, Egeler C, Williams DJ. The effect of needle dimensions and infusion rates on injection pressures in regional anaesthesia needles: a bench-top study. Anaesthesia. 2015 Feb;70(2):183–9.

4. Saporito A, Quadri C, Kloth N, Capdevila X. The effect of rate of injection on injection pressure profiles measured using in-line and needle-tip sensors: an in-vitro study. Anaesthesia. 2019 Jan;74(1):64–8.

5. Benne. Stepper Motor with DRV8825 and Arduino Tutorial (4 Examples) [Internet]. Makerguides.com. 2019 [cited 2023 Jan 5]. Available from: https://www.makerguides.com/drv8825-stepper-motor-driver-arduino-tutorial/

6. Stracciolini A, Hammerberg EM. Acute compartment syndrome of the extremities. In: UpToDate. 2018.

7. Hameln Pharma ltd. Lidocaine Injection BP with Preservative 1% - Summary of Product Characteristics (SmPC) - (emc) [Internet]. 2020 [cited 2023 Jan 5]. Available from: https://www.medicines.org.uk/emc/product/6267/smpc#gref

8. ChemSrc. Lidocaine [Internet]. 2023 [cited 2023 Jan 5]. Available from: https://www.chemsrc.com/en/cas/137-58-6_15385.html

9. Zhang X, Liu J, Guo J, Feng L. A Study of Safety Evaluation Method for Medical Diagnostic Table. Engineering. 2013 Oct 16;05(10):207.

10. Fischer KM, Howell AP. Reusability of autoclaved 3D printed polypropylene compared to a glass filled polypropylene composite. 3D Print Med. 2021 Aug 9;7(1):20.

11. Ou Q, Pei C, Chan Kim S, Abell E, Pui DYH. Evaluation of decontamination methods for commercial and alternative respirator and mask materials – view from filtration aspect. Journal of Aerosol Science. 2020 Dec 1;150:105609.

12. Ipas. Clinical Updates in Reproductive Health [Internet]. 2021 [cited 2023 Feb 24]. Available from: https://www.ipas.org/wp-content/uploads/2021/06/Clinical-Updates-in-Reproductive-Health-CURHE21.pdf

13. Powell B, Kapp N. Validation of instrument reprocessing methods for the Ipas manual vacuum aspiration devices. International Journal of Gynecology & Obstetrics. 2019;147(1):89–95.

14. WomanCare Global. THE Ipas Manual Vacuum Aspiration Technology Product Line Catalogue [Internet]. 2022 [cited 2023 Feb 24]. Available from: https://dktwomancare.org/pdf/Ipas_mva_product_catalogue.pdf

15. Kumar A, Yap WT, Foo SL, Lee TK. Effects of Sterilization Cycles on PEEK for Medical Device Application. Bioengineering. 2018 Mar;5(1):18.

16. Friedericy HJ, van Egmond CW, Vogtländer JG, van der Eijk AC, Jansen FW. Reducing the Environmental Impact of Sterilization Packaging for Surgical Instruments in the Operating Room: A Comparative Life Cycle Assessment of Disposable versus Reusable Systems. Sustainability. 2022 Jan;14(1):430.
